# Multiphoton Absorption Spectra of Channelrhodopsin‑2 via Multiscale Simulation Methods

**DOI:** 10.1021/acs.jctc.5c01719

**Published:** 2026-01-06

**Authors:** David Carrasco-Busturia, Mathieu Linares, Patrick Norman, Jógvan Magnus Haugaard Olsen

**Affiliations:** † Division of Theoretical Chemistry and Biology, 7655 School of Engineering Sciences in Chemistry, Biotechnology and Health, KTH Royal Institute of Technology, Stockholm SE-100 44, Sweden; ‡ PDC Center for High Performance Computing, KTH Royal Institute of Technology, Stockholm SE-100 44, Sweden; § DTU Chemistry, 5205 Technical University of Denmark, Kgs.Lyngby DK-2800, Denmark

## Abstract

Channelrhodopsin-2 (ChR2) is a light-gated ion channel widely used in optogenetics, a technique that enables precise control of neuronal activity by genetically engineering light-sensitive proteins into cell membranes. This protein exists in dimeric form, with each monomer containing a retinal Schiff base (RSB) moiety covalently bonded that undergoes trans–cis isomerization upon light absorption. However, the limited penetration depth of visible light in biological tissues motivates the use of multiphoton-absorption techniques, which enhance tissue penetration, improve focality, and reduce phototoxicity, thereby offering a promising alternative for optogenetic applications. In this paper, we present a fully atomistic multiscale methodology for computing the one-, two-, and three-photon absorption spectra of ChR2, where the protein, lipid bilayer, and solvent are explicitly considered throughout the workflow. This methodology integrates classical molecular mechanics (MM) molecular dynamics (MD), quantum mechanics/molecular mechanics (QM/MM)-MD, and fragment-based polarizable embedding (PE) to derive environment-specific PE potentials from the explicit protein–lipid-solvent environment. The final step in the methodology is to use these potentials to compute accurate spectra via PE-time-dependent density functional theory (PE-TD-DFT). Validation against experimental one-photon absorption spectra demonstrates excellent agreement. For the first time, we report the theoretical two- and three-photon absorption in ChR2, albeit without direct experimental comparison. We compare the multiphoton absorption (MPA) spectra where the two RSB moieties are sampled using classical MD and QM/MM-MD, respectively. The resulting spectral differences are attributed to variations in key structural parameters that we analyze and document.

## Graphical abstract

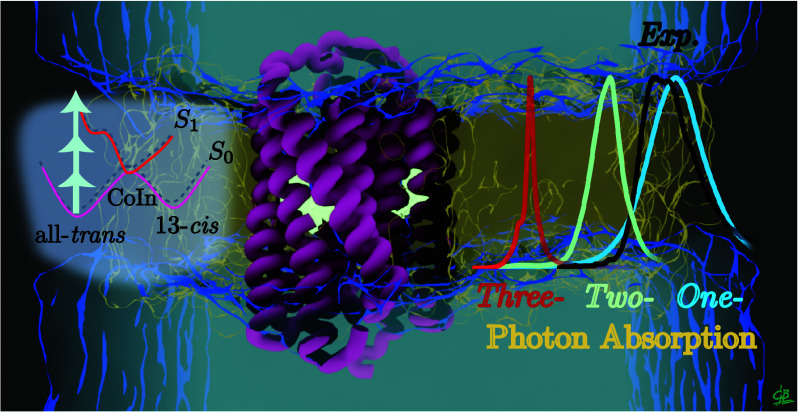

## 1 Introduction

A breakthrough in neuroscience occurred when a technique was developed that allowed the precise control of neuronal activity using light.^1,2^ This technique, termed optogenetics,^3^ employs light-sensitive proteins genetically introduced into neuronal cell membranes to manipulate cellular processes optically. Among the most widely used optogenetic tools is channelrhodopsin-2 (ChR2),^4^ a class of rhodopsin protein composed of seven transmembrane (TM) α-helices, with a retinal Schiff base (RSB) moiety covalently bonded in an internal pocket. The protein exists as a dimer in the cell^5^ which in turn leads to two RSB moieties (Figure 1). ChR2 functions as a light-gated ion channel: upon photon absorption, RSB undergoes a trans–cis isomerization,^5^ triggering the opening of a channel through the cell membrane. The high temporal precision makes channelrhodopsins useful tools for investigating neural circuits involved in memory formation, consolidation, and retrieval.^3^ Consequently, they are useful to better understand the brain, including cognitive decline during aging and neurodegenerative conditions such as Alzheimer’s disease.^6^

**Figure 1:**
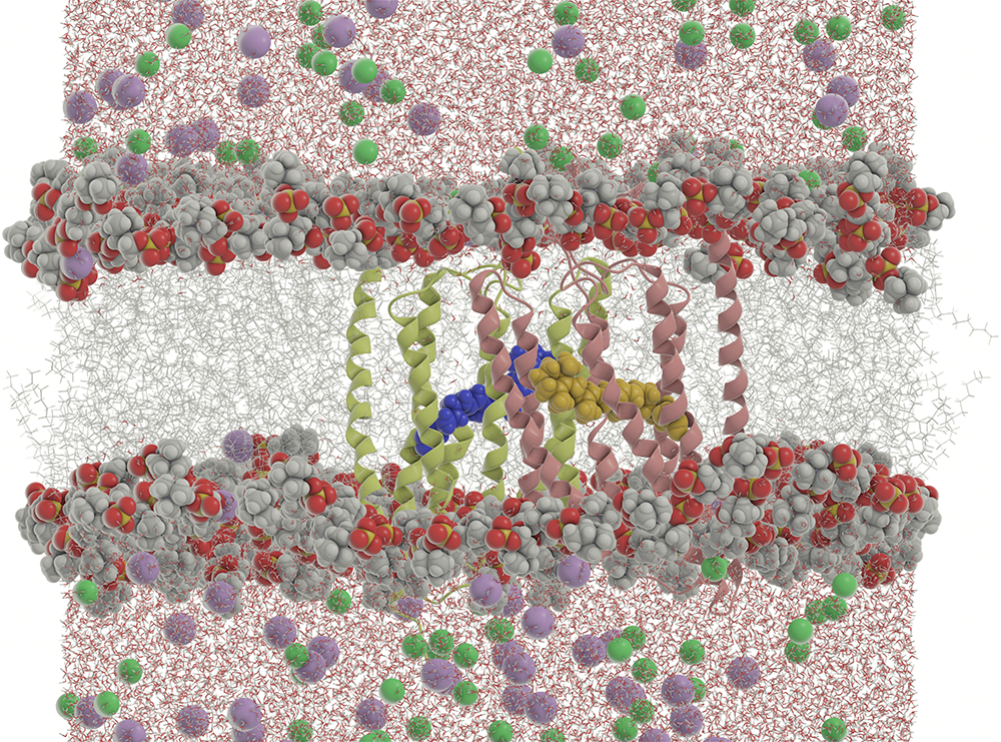
Overview of the channelrhodopsin-2 (ChR2) system studied in this work. The protein exists in the cell as a dimer. Each monomer is composed of seven transmembrane α-helices whose secondary structure is shown in lime and red. Each monomer contains a retinal Schiff base (RSB) moiety, which is shown in blue and yellow, respectively, in van der Waals representation. The 1-palmitoyl-2-oleoyl-*sn*-glycero-3-phosphocholine (POPC) lipid tails are shown in gray stick representation, and lipid heads are shown in van der Waals representation. Na^+^ and Cl^–^ ions are shown in purple and green, respectively, and water molecules are also shown.

Despite its advantages, *in vivo* optogenetic applications face challenges due to the scattering of visible light by biological tissue, which limits the penetration depth.^7^ Multiphoton absorption (MPA) provides a promising alternative by allowing the RSB moiety to be excited through the simultaneous absorption of multiple lower-energy photons. The use of longer wavelengths mitigates scattering effects, increases tissue penetration, enhances focality, reduces phototoxicity (thus reducing cell damage), and minimizes competition with hemoglobin.^8,9^ MPA cross sections, which quantify the probabilities of absorption, are crucial to identify how readily an opsin will respond to multiphoton illumination.^10^ However, their experimental determination in biomolecules is complicated by factors such as uncertainties in determining protein concentration and the occurrence of spurious nonlinear optical processes.^11^ Reliable computational methods are therefore indispensable. Accurately modeling the spectra of rhodopsins requires thorough molecular dynamics (MD) sampling across a wide range of configurations to obtain reliable spectroscopic properties, as has been demonstrated in studies such as those conducted for bovine rhodopsin.^12^ Given that light absorption is a fundamentally quantum-mechanical (QM) process, it necessitates a QM description. However, the computational cost required to investigate transmembrane proteins at this level of theory remains prohibitively expensive. To address this challenge, multiscale approaches and, in particular, hybrid quantum mechanics/molecular mechanics (QM/MM) methods provide a practical and effective solution.^13–20^ Computational atomistic studies of ChR2 were hindered until its structure was resolved in 2017.^5^ Prior to this, classical molecular mechanics (MM)-MD studies of the channel opening mechanism have primarily focused on C1C2, a chimeric structure combining ChR1 and ChR2.^21^ Once the ChR2 structure was resolved, nonadiabatic dynamics simulations provided insights into the RSB photoisomerization mechanism in both wild-type^22^ and mutant variants.^23^ Classical MM-MD investigations have been conducted in conjunction with QM/MM-MD force matching to study the initial steps of the pore opening and influx of water molecules.^24^ So far, all these investigations have been conducted assuming a single nonbranched photocycle model. However, recent experiments in electrophysiology, time-resolved step-scan FTIR, and Raman spectroscopy reveal a two-photocycle model, a branched model with two open and two closed states.^25^ In addition to photoisomerization studies, computational studies have investigated the effect of mutations on spectroscopic properties, such as variations in absorption wavelengths in bovine rhodopsin mutants,^26^ or the influence of mutations on C1C2 two-photon absorption cross sections.^27^ To our knowledge, there has been no atomistic computational investigation of the MPA processes in ChR2.

In this work, we propose a multiscale strategy to compute the one-, two-, and three-photon absorption spectra of ChR2, using an explicit all-atom protein–lipid-solvent environment (i.e., no implicit-solvent models) throughout the MM-MD, QM/MM-MD sampling and PE-TD-DFT calculations. We link spectral variations to structural differences in the two RSB moieties. We describe the different computational methodologies employed at each stage of the workflow and, in particular, discuss in detail the treatment of the environment through different fragment-based approaches, with special emphasis on the several approximations used to account for the contributions from the environment.

## 2 Theoretical Methods

The multiscale strategy followed in this work is comprised of five stages, further explained in each of the subsections of this section:

System preparation
MM-MD equilibration and production
QM/MM-MD configurational sampling
Fragment-based polarizable embedding (PE) potential
PE-TD-DFT-based MPA spectra

The multiscale strategy followed in this work is summarized in Figure 2 and is further explained in each of the subsections of this section. It comprises five stages: after a system preparation, we perform MM-MD equilibration and production runs, followed by QM/MM-MD configurational sampling, and TD-DFT for the calculation of the MPA spectra combined with fragment-based polarizable embedding for describing the MM environment.

**Figure 2:**
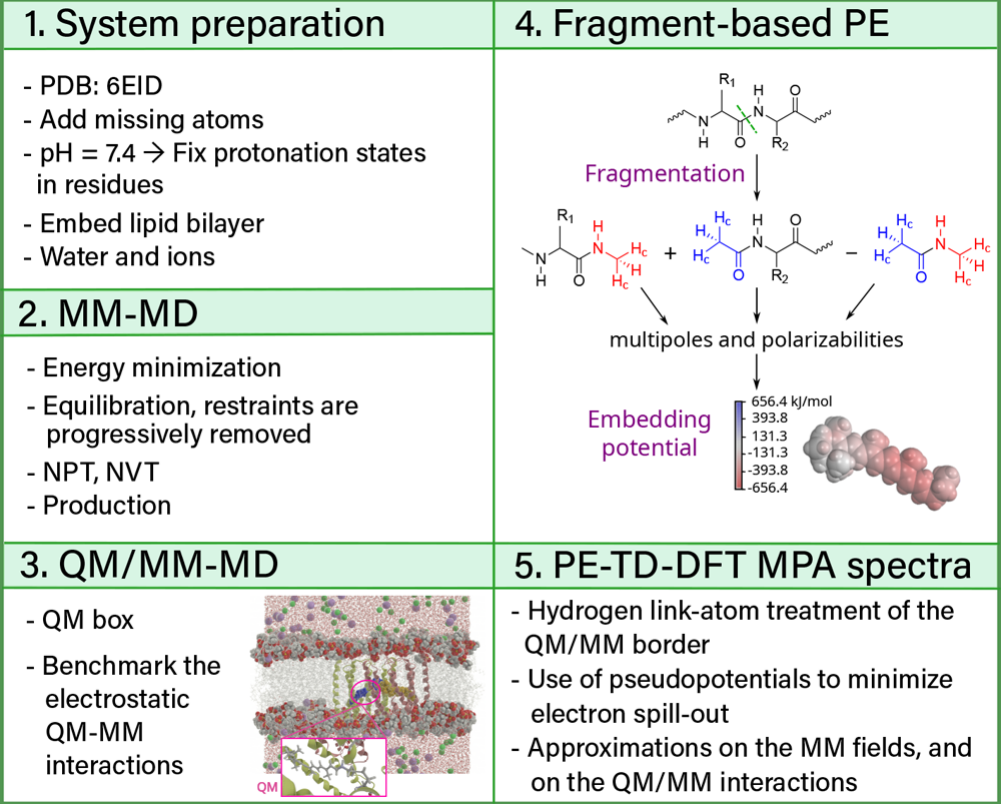
General workflow followed in this work.

### 2.1 System Preparation

The crystal structure of wild-type ChR2 (PDB ID: 6EID^5^) was used as a starting point. Chain B and cocrystallized molecules and phosphate ions were removed. The dimeric form of ChR2 was generated in VMD^28^ using the BIOMT transformation matrix written in the PDB file. A protein preparation workflow was followed as implemented in Maestro^29^ to take it from its raw state to a state where the protein is properly prepared for MD calculations. Second, missing atoms from residues were added. The hydrogen bonding network was optimized by reorienting hydroxyl, thiol groups, water molecules, the amide groups of asparagine (Asn) and glutamine (Gln), and the imidazole rings of histidine (His). The protonation states of His, aspartic acid (Asp), and glutamic acid (Glu) were assigned based on the local environment of each residue at pH 7.4 as determined from the p*K*_a_ prediction by the PROPKA3 method.^30,31^ An energy minimization was performed with the OPLS4 force field,^32^ where all atoms were minimized until the RMSD of heavy atoms relative to the starting structure exceeded the threshold criteria of 0.3 Å. The RSB, as well as the Asp, Glu, Lys, and Arg residues, were in their ionic form in both chains, except for Asp156, Glu90, and Glu101, which were protonated. All His residues are in their neutral state, with the proton in either N_ϵ_ (residues 114, 134, 201, 265 in chain A and residues 134, 191, 201, 265 in chain B) or in N_δ_ (residues 191, 249, 272, 274, 278 in chain A and residues 114, 249, 272, 274, 278 in chain B). The CHARMM-GUI membrane builder^33^ was then used to add disulfide bonds between Cys36 (chain A) and Cys34 (chain B), and between Cys34 (chain A) and Cys36 (chain B). The protein was then embedded in a 1-palmitoyl- 2-oleoyl-*sn*-glycero-3-phosphocholine (POPC) bilayer solvated by water and a concentration of 0.15 M NaCl. The box size of the system was 120 nm × 120 nm × 151 nm, with *z* being the cross-membrane direction.

### 2.2 MM-MD Equilibration and Production

All MM-MD simulations were performed with GROMACS 2019.4 ^34^ using the CHARMM36 force field.^35^ Most of the following MD settings were used as suggested by CHARMM-GUI. The short-range van der Waals and Coulomb cutoff distances were both set to 1.2 nm. The system was simulated using periodic boundary conditions. Particle mesh Ewald^36^ was used to calculate long-range electrostatic interactions.

Rigid holonomic constraints were used on covalent bonds involving hydrogen atoms using the LINCS algorithm^37^ while SETTLE^38^ constraints in rigid TIP3P water were applied. First, an energy minimization was performed using the steepest descent algorithm with a maximum force tolerance of 1000 kJ·mol^–1^·nm^–1^ and a maximum of 5000 steps.

Several potentials were used for imposing restraints on the motion of the POPC bilayer as well as on both protein chains. These restraints were progressively removed during six equilibration steps (more information in the Supporting Information).

After the sixth equilibration run, an NPT production run was performed without restraints for 50 ns and with a 2 fs time step using the Nosé–Hoover thermostat^39^ and Parrinello–Rahman barostat^40^ and the default leapfrog algorithm for integrating Newton’s equations of motion. The next step of the workflow is QM/MM-MD, which in the MiMiC framework is only implemented in the NVT ensemble (more details in Section 2.3). In that stage, we also remove rigid holonomic constraints on covalent bonds to hydrogen atoms (except water), which necessitates a shorter integration time step. To mirror these conditions and ensure a smooth transition between MM-MD and QM/MM-MD, we conducted an intermediate MM-MD NVT equilibration, which was performed in two steps: First, a 50 ns NVT run at the same 2 fs time step, using the Nosé–Hoover thermostat,^39^ starting from the last configuration of the previous NPT trajectory. Second, a further 25 ns NVT run at 1 fs time step, where rigid holonomic constraints on covalent bonds involving hydrogen atoms were removed, while SETTLE^38^ constraints in rigid TIP3P water were maintained.

### 2.3 QM/MM-MD Configurational Sampling

QM/MM-MD simulations were conducted with the MiMiC multiscale modeling framework^41–43^ using CPMD 4.3 ^44^ and GROMACS 2021.6 ^34,45^ for the QM and MM subsystems, respectively.

The system is composed of 204,687 atoms where the RSB moiety, formed by 63 atoms, is the QM subsystem. This subsystem is modeled using the BLYP exchange-correlation (XC) functional^46^ including Grimme’s D3 correction^47^ using the new driver for the calculation of the XC contribution as implemented in the CPMD code.^48^ Troullier–Martins norm-conserving pseudopotentials were used to model core electrons.^49^ In our QM/MM-MD simulations with CPMD code as the QM client, which uses a plane-wave basis, we need to ensure isolated system conditions for the QM subsystem, which is done by solving the Poisson’s equation. The Poisson solver that we use in CPMD is the Martyna–Tuckerman,^50^ which requires the charge density to be (numerically) zero at the border of the QM box.^44^ In practice and for larger molecules (such as our RSB), the Martyna–Tuckerman solver requires that the size of the QM simulation box be twice the size of the charge density.^44^ This results in an orthorhombic QM unit cell with dimensions of *a* = 92.60 a.u., and *b* = *c* = 62.58 a.u. The MM subsystem is described by the same force field as in the classical MD simulations, and all classical interactions were computed using the same settings. At each step, the QM subsystem is centered, and molecules are made whole. The electrostatic QM-MM interactions are calculated without periodic repetition and using an efficient coupling scheme.^41^ Here, all interactions involving MM fragments or atoms within a specified cutoff are computed exactly using the QM electron density directly whereas the interactions for MM fragments or atoms located further from that cutoff are calculated via a multipole expansion of the QM electrostatic potential. In this work, the cutoff itself is defined by the distance between the centroid of the QM subsystem and the individual atoms in the MM environment. The dependency on the short-range cutoffs and multipole orders of the energy and forces was investigated, as shown in Section 4. The QM boundary atom is the C_α_ atom of the RSB moiety (Figure 3). This atom is described by a monovalent pseudopotential.^51^ The MiMiCPy Python package^52^ was used to prepare the input files.

**Figure 3:**
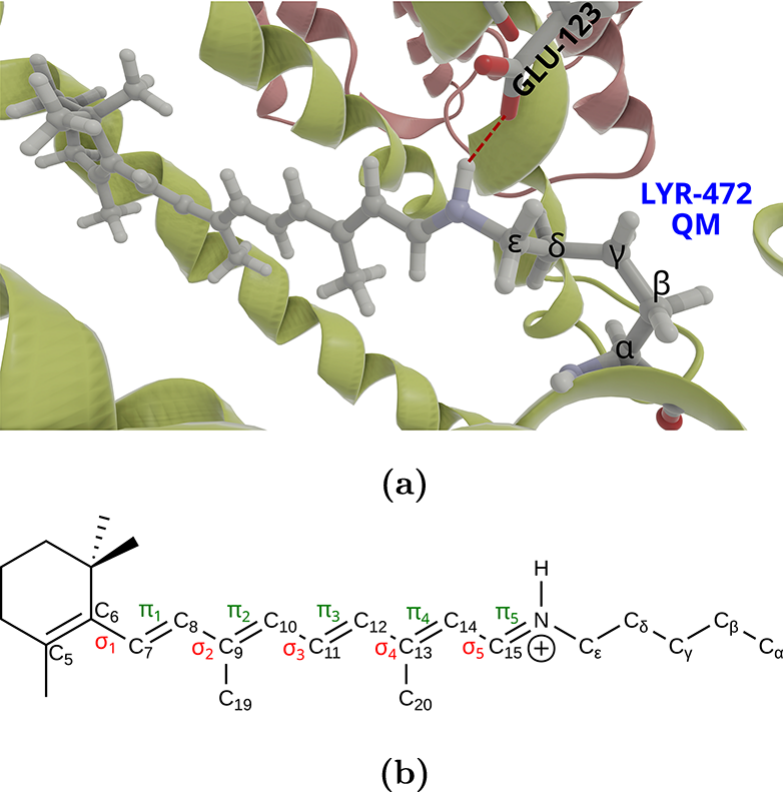
All-*trans* isomer of ChR2 and its environment (a) and the chemical structure of the moiety (b), where each of the bonds studied in the main text is labeled as σ_*i*_ or π_*i*_, where *i* = 1–5.

The DIIS method^53–55^ was utilized to accelerate the self-consistent field (SCF) process. A convergence threshold was set to 10^–5^ a.u. with respect to the largest component of the electronic gradient. To further accelerate convergence, the initial wave function guess at each MD step is based on an extrapolation from the preceding five steps.^56^ A Born–Oppenheimer (BO) QM/MM-MD equilibration run was performed in the NVT ensemble for 5 ps, corresponding to 10,000 steps using a time step of ≈ 0.48 fs (20.0 a.u.). The temperature was maintained at 303.15 K using the Berendsen thermostat^57^ and a time constant of 5000 a.u. After, we continued with a 45 ps NVT production run using four Nosé–Hoover thermostats^58–60^ each having its own chain^39^ and a frequency of 5000 cm^–1^: *i*) the QM subsystem, *ii*) the membrane, *iii*) the protein, and *iv*) water and ions.

### 2.4 Fragment-Based Polarizable Embedding

To obtain accurate spectra, snapshots from the QM/MM-MD trajectory are extracted, and the environment composed of proteins, lipid bilayer, water, and solvation ions will be described by a polarizable model. Unlike in electrostatic embedding, where the MM environment is represented by point charges that interact electrostatically with the QM electron density, in more advanced polarizable models, like in the fragment-based polarizable embedding (PE) method, the environment is described by atom-centered multipoles and polarizabilities. The multipoles describe the static charge distributions of fragments in the environment, while polarizabilities are used to model their induced charge distributions. The polarizabilities generate induced dipoles in response to the electric fields from the electrons and nuclei in the QM subsystem, as well as from both the static multipoles and the induced dipoles from the other fragments in the environment. This approach ensures a fully self-consistent description of the environment polarization.^61,62^

### 2.5 The Embedding Potential

Accurate spectroscopic properties rely on the quality of the embedding potential.^63–66^ In the fragment-based PE method, the classical embedding potential parameters, i.e., multipoles and polarizabilities, are derived directly from QM calculations on the individual fragments that make up the environment.^67,68^ In this work, the biological system is divided into four regions: water molecules within 20 Å of the RSB core (which include water molecules inside the ion channel), the rest of water molecules and ions (both above and below the membrane), the lipid bilayer, and the protein. The embedding parameters for the water molecules within 20 Å of the RSB core were derived for each water molecule in isolation based on QM calculations. A more advanced fragmentation method is needed for the protein. For this, the molecular fractionation with conjugate caps (MFCC) method was employed, where the system is divided into small fragments, each capped with groups of atoms from neighboring fragments (see Figure 2, Step 3). Two neighboring caps are merged to form a so-called concap fragment to remove the double counting introduced by the capping.^64,69,70^ The fragmentation and generation of embedding potential parameters (LoProp-based^71^ atom-centered charges, dipoles, quadrupoles, and anisotropic dipole–dipole polarizabilities) were facilitated using PyFraME.^72^ The fragment calculations were performed at the CAM-B3LYP^73^/loprop-6-31+G* level of theory (where loprop-6-31+G* is an ANO-type recontraction of 6-31+G*) using the Dalton program^74^ and the LoProp for Dalton Python package.^75^ Due to the system being quite large, the MFCC approach was adopted only for the protein. The lipids were modeled using parameters from the averaged lipid embedding potential (ALEP).^76^ Ions and water molecules beyond 20 Å from the RSB core were modeled with averaged solvent embedding potential (SEP) parameters.^77^ Both ALEP and SEP sets of parameters consist of averaged atom-centered charges and isotropic dipole–dipole polarizabilities. In the following, this approach is labeled as ***mfcc*** (see Figure 4).

**Figure 4:**
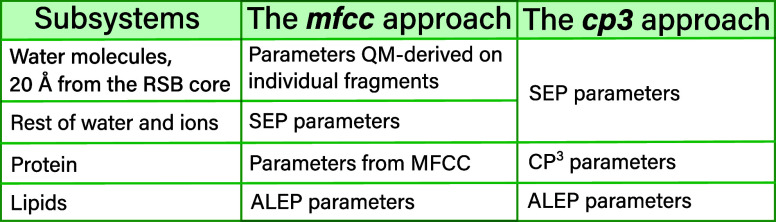
***cp3*** and ***mfcc*** approaches used for the different subsystems of the environment to derive the embedding potential.

To further reduce the computational cost, a second approach was to use ALEP for the lipids, SEP for the solvent, and to also describe the protein with averaged parameters from the cost-effective polarizable protein potential (CP^3^),^78^ which contains atom-centered charges and isotropic dipole–dipole polarizabilities for all of the standard amino acids. Since there are no such parameters available for the retinal part of the RSB moiety (i.e., the β-ionone ring and atoms C_7_ to C_15_ and its hydrogens), we derived those parameters ad hoc in the following way: We use PyFraME^72^ to cut the N–C_15_ bond and saturate the missing valence with a hydrogen atom and treat this fragment at the CAM-B3LYP^73^/loprop-6-31+G* level of theory. This approach is labeled ***cp3*** in the following (see Figure 4).

The performance and effect of both ***mfcc*** and ***cp3*** approaches on the spectroscopic properties will be shown in Section 4.2.

### 2.6 Electron Spill-Out

In classical embedding, such as PE, there is a lack of Pauli repulsion between the QM and MM subsystems, which can lead to electron density leaking into the environment. This is an unphysical effect known as electron spill-out. This effect is particularly pronounced with basis sets that include diffuse functions.^79–82^ Although removing these functions may mitigate the electron spill-out, it risks compromising an accurate description of the excited states.^83^ An effective solution to electron spill-out involves strategically placing pseudopotentials (PPs) at the positions of the MM atoms, which mimics the Pauli repulsion missing in the PE model. In this study, we placed PPs on all atoms belonging to fragments (excluding lipids) that have at least one atom within 6 Å from any atom of the RSB chromophore. We exclude placing PPs on the C_α_ atom to prevent spurious interactions with the hydrogen link atom.^83^ The hydrogen link atom is described using the STO-3G basis set to further minimize the risk of electron spill-out at the boundary. The impact of placing PPs on the computed excitation energies and TPA cross sections is quantified in Section 4.2.

### 2.7 Approximations in PE

PE methods are substantially more computationally demanding than nonpolarizable QM/MM methods due to the need to solve polarization equations at each SCF iteration. Formally, this computational cost scales quadratically with the number of polarizable sites, making these methods particularly expensive for large systems containing on the order of 10^5^ atoms. Furthermore, for large quantum subsystems, the cost of calculating electronic electric fields on all polarizable sites in the environment, which is also needed in every SCF iteration, and to compute Fock matrix contributions, further increases the computational demands.^84^ This work employs several approximations to reduce the computational cost for the relatively large ChR2 system. These approximations affect the calculation of MM fields and the QM/MM interactions.

#### 2.7.1 Approximations on the MM Fields

One of the first approximations we introduce in this study concerns the evaluation of the MM fields. The fast multipole method (*FMM*) recursively subdivides the MM subsystem into hierarchical boxes using an octree.^85^ Interactions between *near* boxes are evaluated directly, while interactions between *well-separated* boxes are approximated via truncated multipole expansions, thereby replacing many site–site interactions with a smaller number of box–box operations, which can reduce the formal scaling of solving the polarization equations from quadratic to linear for sufficiently large systems. The accuracy and speed of *FMM* are controlled by two parameters: the multipole acceptance criteria (θ, which determines whether two boxes, A and B can be considered far separated), and the multipole order (*p*, which controls the order of the multipole expansion in *FMM*). More details can be found in refs 85 and 86. The effect of these parameters on the calculations is presented in Section 4.2.

#### 2.7.2 Approximations on the QM/MM Interactions

In this work, we have investigated the acceleration of the QM/MM interactions through two strategies, i.e., *single-center multipole expansion* and *electrostatic potential fitting (ESPF)*: *i)* In the *single-center* strategy, the molecular multipoles of the QM subsystem are computed and used to compute the electric field on polarizable sites outside a defined cutoff radius.^41,42,87^ *ii)* In the *ESPF* strategy, atom-centered multipoles fitted to reproduce the electrostatic potential are used to compute the field.^88–93^ More details about the theory and implementation of both approaches can be found in ref 84. The effect of the order *p* of the multipoles as well as the cutoff radius *R*_exact_, beyond which the approximate coupling is used, will be shown in Section 4.2.

## 3 Spectra

For each snapshot, the fragment-based PE potential parameters are derived as described above and used to calculate the MPA spectra via PE-TD-DFT using the Dalton program^74^ together with the PE library.^94^

### 3.1 One-Photon Absorption

The one-photon absorption spectrum will be presented as the molar absorption coefficient ϵ­(ω), which is calculated from the excitation energies and oscillator strengths:

1 $$\epsilon \left(\omega \right)=\frac{e^{2}\pi N_{A}}{2\ln\left(10\right)\epsilon_{0}nm_{e}c}\underset{i}{\sum }\frac{\omega f_{i}}{\omega_{i}}g\left(\omega ,\omega_{i},\gamma_{i}\right)$$

where *N*_*A*_ is the Avogadro constant, *e* is the elementary charge, *m*_*e*_ is the electron mass, *c* is the speed of light, ϵ_0_ is the vacuum permittivity, *n* is the refractive index (here set to 1), *f*_*i*_ is the calculated oscillator strength of the *i*th transition, ω_*i*_ is the *i*th transition angular frequency, and *g* is a line-broadening function, typically a Lorentzian described with a γ_*i*_ parameter corresponding to the half-width at half-maximum (HWHM).^95^

### 3.2 Two- and Three-Photon Absorption

The two-photon (TPA) and three-photon (3PA) absorption spectra are calculated as^96^

2 $$\sigma^{\mathrm{TPA}}\left(\omega \right)=\frac{8\pi^{3}}{c_{0}^{2}\hbar^{2}}e^{4}\underset{i}{\sum }\omega^{2}\delta_{i}^{\mathrm{TPA}}g\left(2\omega ,\omega_{i},\gamma_{i}\right)$$

3 $$\sigma^{\mathrm{3PA}}\left(\omega \right)=\frac{16\pi^{4}}{c_{0}^{3}\hbar^{3}}e^{6}\underset{i}{\sum }\omega^{3}\delta_{i}^{\mathrm{3PA}}g\left(3\omega ,\omega_{i},\gamma_{i}\right)$$

where σ and δ_*i*_ are, respectively, the cross sections and *i*th electronic transition strengths. Similar to eq 1, a line-broadening function *g* is applied. Cross sections are usually given in the cgs unit system, that being $\frac{{\mathrm{cm}}^{4}\cdot s}{\mathrm{photon}}$ and $\frac{{\mathrm{cm}}^{6}\cdot s^{2}}{{\mathrm{photon}}^{2}}$ for TPA and 3PA, respectively. For the particular case of TPA, cross sections are typically given in Göppert-Mayer units (GM), with $1\mathrm{GM}=10^{-50}\frac{{\mathrm{cm}}^{4}\cdot s}{\mathrm{photon}}$ . Equations 2 and 3 yield the TPA and 3PA spectra, respectively, in GM and $\frac{{\mathrm{cm}}^{6}\cdot s^{2}}{{\mathrm{photon}}^{2}}$ units, as

4 $$\sigma^{\mathrm{TPA}}\left(\omega \right)=2.505472\times 10^{-2}\underset{i}{\sum }\omega^{2}\delta_{i}^{\mathrm{TPA}}g\left(2\omega ,\omega_{i},\gamma_{i}\right)$$

5 $$\sigma^{\mathrm{3PA}}\left(\omega \right)=7.781292\times 10^{-7}\underset{i}{\sum }\omega^{3}\delta_{i}^{\mathrm{3PA}}g\left(3\omega ,\omega_{i},\gamma_{i}\right)$$

## 4 Results and Discussion

### 4.1 QM/MM-MD

To determine the optimal treatment of the electrostatic QM/MM interactions during the MD simulations, we benchmarked various short-range cutoff distances and multipole orders (c.f. Section 2.3). Figure 5a,b shows the errors in the total energy and in the maximum Cartesian force component, respectively, for each combination of short-range (SR) cutoff and multipole order (MO), using as a reference a full short-range calculation (i.e., a direct calculation that evaluates all pairwise electrostatic interactions explicitly without cutoffs or MO expansion). We evaluated numerical convergence against the full short-range reference. At an SR cutoff of 65 a.u. and MO of 8, we obtain |Δ*E*_tot_| around 9 × 10^–6^ a.u. and |Δ*f*_max_| around 2 × 10^–6^ a.u. We regard these errors as sufficiently small for the present work and therefore adopted these settings for production simulations. Additionally, we evaluated the convergence of the energy and maximum Cartesian force component with respect to the plane-wave cutoff energy *E*_cut_ at fixed (SR, MO) = (65, 8) by varying *E*_cut_ from 50 to 110 Ry, and using the 120 Ry calculation as reference (Figure 5c,d). Both |Δ*E*_tot_| and |Δ*f*_max_| decrease and reach 2 × 10^–2^ a.u. and 5 × 10^–4^ a.u., respectively, at *E*_cut_ = 100 Ry, which corresponds to a real-space grid of 600 × 400 × 400.

**Figure 5:**
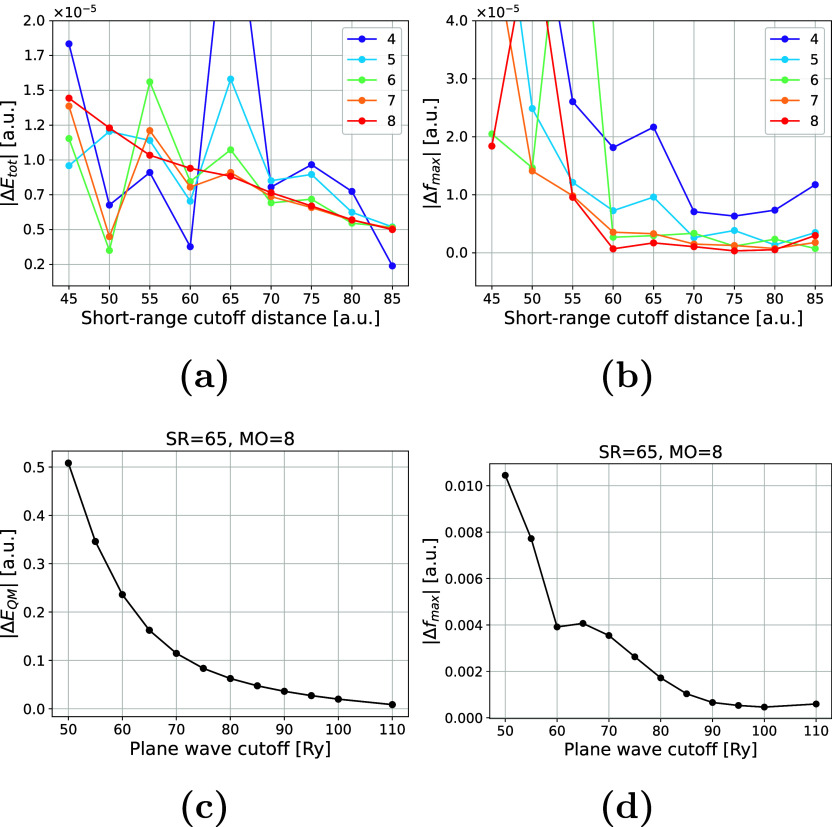
Convergence of the total energy (a) and forces (b) with respect to short-range cutoff distances and the order of the multipole expansion, using the full short-range coupling as a reference. In the lower panel, we demonstrate the convergence of the QM energy (c) and forces (d) with respect to the plane wave cutoff, using a calculation with a 120 Ry cutoff energy as a reference, and using a fixed short-range cutoff (SR) and multipole order (MO) of 65 and 8, respectively. All points in (a–d) correspond to the absolute errors in the energies and the absolute errors in the maximum Cartesian force component.

Figure 6 presents the time-evolution of energy and temperature during the 5 ps +45 ps QM/MM-MD simulations. The total energy is shown in Figure 6a. Figure 6b shows the Kohn–Sham energy of the QM region, and Figure 6c presents the temperature of the QM and MM regions.

**Figure 6:**
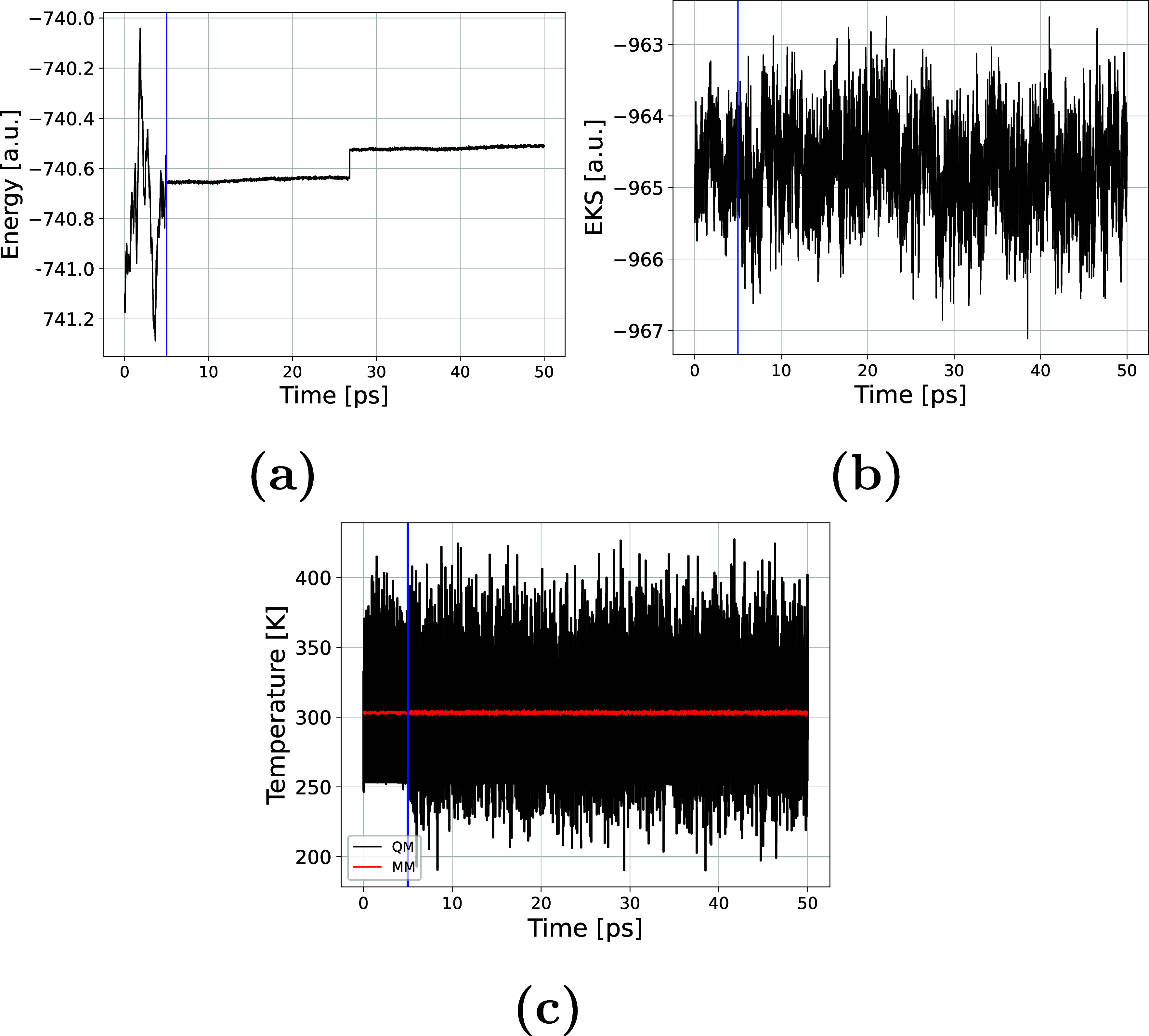
Time evolution of the total energy (a), the Kohn–Sham energy (b), and temperature (c) during the QM/MM-MD simulation. The first 5 ps of the simulation were conducted using a Berendsen thermostat, followed by 45 ps using four separate Nosé–Hoover thermostats. The vertical blue line at 5 ps marks the thermostat switch.

#### 4.1.1 Parallel Scaling and Performance

In QM/MM-MD, the wall time per MD step is dominated by the QM code; however, efficient communication between the QM and MM codes is critical.^20^ The MiMiC framework addresses this with a client-server, MPMD design and an efficient communication library, enabling concurrent execution of the QM and MM programs on separate resources and network-based communication; this avoids file I/O operations, and keeps subsystem-interaction calculations highly parallel and efficient.^43^ In our setup (CPMD as the QM client), we combine MPI with OpenMP multithreading, and the best performance is obtained when both are used.^42^ We evaluated hybrid MPI/OpenMP scaling by measuring the average time per MD step (last 10 steps of a 20-step run; Berendsen thermostat; Δ*t* = 0.48 fs), while varying node count and MPI process/OpenMP thread layout (Figure 7, upper panel). As shown in the lower panel of Figure 7, the time per MD step decreases to ≈ 13 s per MD step at 75 nodes with 8 MPI processes per node and 5 OpenMP threads per MPI process. This configuration provided the best performance and was used for the production simulations. During the equilibrated Nosé–Hoover trajectory, a performance of 5.32 ps/day was achieved.

**Figure 7:**
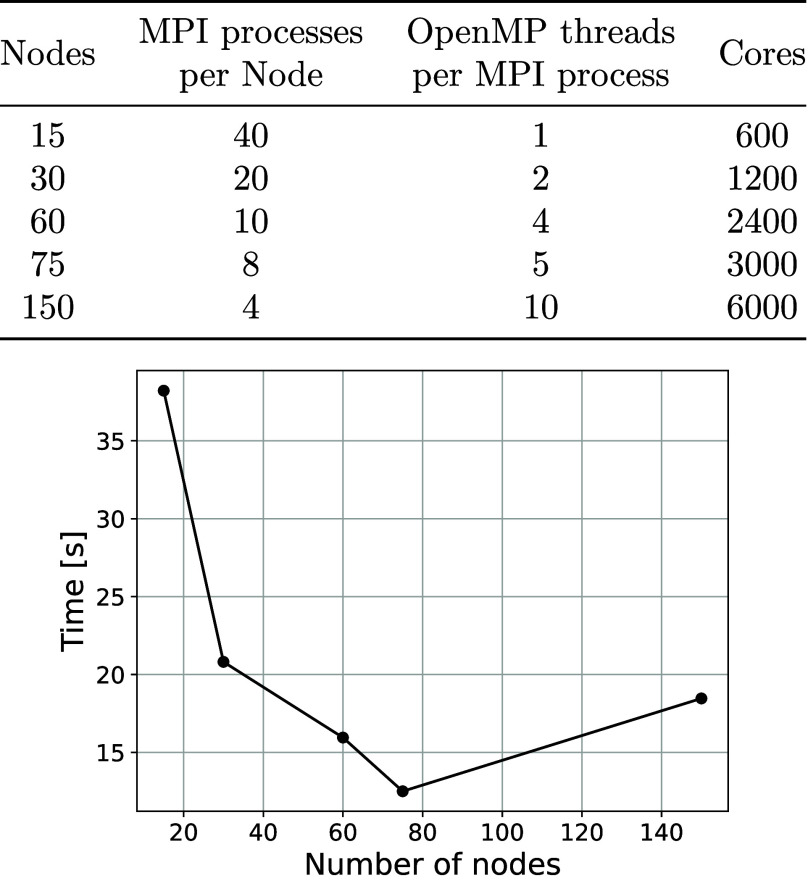
Hybrid MPI/OpenMP scaling of the QM/MM-MD run. Upper panel: MPI process/OpenMP thread layout with the number of cores and nodes. In all cases, there is 1 plane per MPI process. Lower panel: time per MD step. Scaling study was carried out on the CPU module of the Computerome cluster, consisting of nodes with 2 × Intel Xeon Gold 6230 CPU (2 NUMA nodes, each composed of 20 cores).

### 4.2 Fragment-Based Polarizable Embedding

To optimize computational efficiency without sacrificing accuracy, we first introduced the *FMM* approximation for the MM fields, which reduces the formal scaling of the polarization solve from quadratic to linear for large systems.^85^ Within *FMM*, the multipole acceptance criterion (θ), and the multipole expansion order (*p*) control the approximation; higher θ and lower *p* are more approximate (c.f. Section 2.7.1). We performed a comparison of the default settings (θ = 0.4, *p* = 6) with a slightly looser choice (θ = 0.5, *p* = 5). Loosening the settings changed the first three excitation energies by at most 2 × 10^–4^ eV, the oscillator strengths by ≤10^–3^ (≤0.3%), and the two-photon cross sections by ≤20 GM (≤0.36%), while reducing the TPA wall time from 13.4 h to 12.4 h. We therefore adopted θ = 0.5, *p* = 5 in all subsequent calculations (see Table S2 for full data).

Regarding the approximations on the QM/MM interactions, two parameters control both the *single-center* multipole expansion and *ESPF* schemes (c.f. Section 2.7.2): The multipole order in the QM expansion (*p*), and the distance within which the exact QM/MM interaction is used (*R*_exact_). For the *single-center* expansion, a relatively large (*R*_exact_) is needed because of its limited radius of convergence.^97–99^ Taking the *single-center* setup with *p* = 8 and *R*_exact_ = 65 a.u. as our reference based on the investigation in Section 4.1, we reduced *R*_exact_ to 40 a.u. which left the first three excitation energies, oscillator strengths unchanged to 10^–4^, and to the last printed digit for the TPA cross-section. The TPA wall time was reduced from 4.6 h to 4.2 h. Switching to *ESPF* with *p* = 0 and *R*_exact_ = 10 changed excitation energies by at most 6 × 10^–4^ eV, oscillator strengths by ≤2 × 10^–4^ (≤0.3%), and TPA cross sections by ≤10 GM (≤0.24%), while reducing the TPA wall time further to 3.3 hours. On this basis, we adopted *ESPF* (*p* = 0 and *R*_exact_ = 10) for production calculations (full data in Table S3).

We calculated embedding potentials for both ***mfcc*** and ***cp3*** approaches. Thereafter, excitation energies, oscillator strengths, and TPA cross sections are obtained with PE-TD-DFT using CAM-B3LYP^73^ with the Dalton program^74^ and the PE library.^94^ We included EEF^100^ effects and appropriate approximations (both for *FMM* and QM/MM interactions). The covalent bond crossing the quantum-classical border is treated as follows: The C_α_–C_β_ bond is cut (Figure 3), and the dangling bond is saturated with a hydrogen link atom. All charges in the environment that are within 0.8 Å of a nucleus in the quantum subsystem are distributed to the three nearest sites in the environment, while all other parameters are removed.^68^ To minimize the effect of electron spill-out, pseudopotentials are placed on atoms as described in Section 2.6. For the PE-TD-DFT calculation, several basis sets were evaluated, namely, 6-31+G*, cc-pVDZ, aug-cc-pVDZ, pcseg-1, aug-pcseg-1, pcseg-2 and aug-pcseg-2 ^101^ for both ***mfcc*** and ***cp3*** approaches. The first three excitation energies and TPA cross-section differences are shown in Figure 8a and b, respectively, where the reference is the ***mfcc*** approach with aug-pcseg-2 basis set. The ***cp3*** approach and aug-pcseg-1 basis set yields Δ*E*_exc_ of 0.02, 0.03, and 0.01 eV (about 0.75%, 0.62% and 0.11% shifts) for the first, second, and third excitation energies, respectively. The corresponding differences in σ^TPA^ are 11, 100, and 660 GM (about 22%, 15%, and 15% shifts) for the first, second, and third excitation energies, respectively. Timings for each basis set are shown in Figure 8c for TPA calculations run on 160 MPI processes across 4 nodes (note that the TPA data point for ***mfcc*** with aug-pcseg-1 basis set was a faulty calculation and is therefore not reported). For ***cp3***, the reported time is the PE-TD-DFT wall-clock time. For ***mfcc***, the plotted time equals the PE-TD-DFT wall-clock time plus the one-off fragment-based calculations required to assemble the embedding potential (cf. Section 2.5). The fragment-based calculations wall times sum up to 10.1 hours and are added as a constant overhead to each ***mfcc*** TPA timing. This overhead is evaluated once for a given environment and is independent of the basis set used in the subsequent PE-TD-DFT step and can be reused for all property calculations. Moreover, the fragment calculations are embarrassingly parallel, and thus using more computational resources will reduce the wall time correspondingly. We therefore adopt ***cp3*** with the aug-pcseg-1 basis set hereafter, as it offers the best cost-accuracy balance with results comparable to aug-pcseg-2. Lastly, the effect of placing PPs on the MM atoms has also been investigated. We compared the effect of placing PPs on the MM atoms (as described in Section 2.6) and not using PPs at all. We performed this analysis using the embedding potential from both ***cp3*** and ***mfcc*** approaches (CAM-B3LYP and aug-pcseg-1 basis set). For an embedding potential coming from ***cp3*** approach, we get a Δ*E*_exc_ of 0.04, 0.07, and 0.02 eV (about 1.5%, 1.7%, and 0.5% shifts) for the first, second, and third excitation energies, respectively. The corresponding differences in σ^TPA^ are 17, 59, and 560 GM (about 26%, 8%, and 11% shifts) for the first, second, and third excitation energies, respectively, indicating a modest but non-negligible effect (full data in Table S4). In contrast, when the embedding potential comes from the ***mfcc*** approach, the difference between using PPs and not using those is much more pronounced: Δ*E*_exc_ of 0.61, 1.5, and 1.6 eV (about 23%, 37% and 37% shifts) for the first, second, and third excitation energies, respectively, and the corresponding differences in σ^TPA^ are 50, 617, and 4319 GM (about 95%, 89%, and 99% shifts, full data in Table S5). The stronger sensitivity observed for the ***mfcc*** approach could be attributed to the higher accuracy and higher degree of sophistication of this potential (e.g., a more detailed description of the environment given by higher order multipoles and anisotropic polarizabilities), which could consequently lead to more pronounced electron spill-out effects. Given these observations, we employ PPs in all production calculations to minimize the effect of electron spill-out and obtain reliable spectroscopic properties. The relevant scripts and all input files necessary to reproduce the results presented in this paper can be found in ref 102. The relevant molecular dynamics trajectories can be found in ref 103.

**Figure 8:**
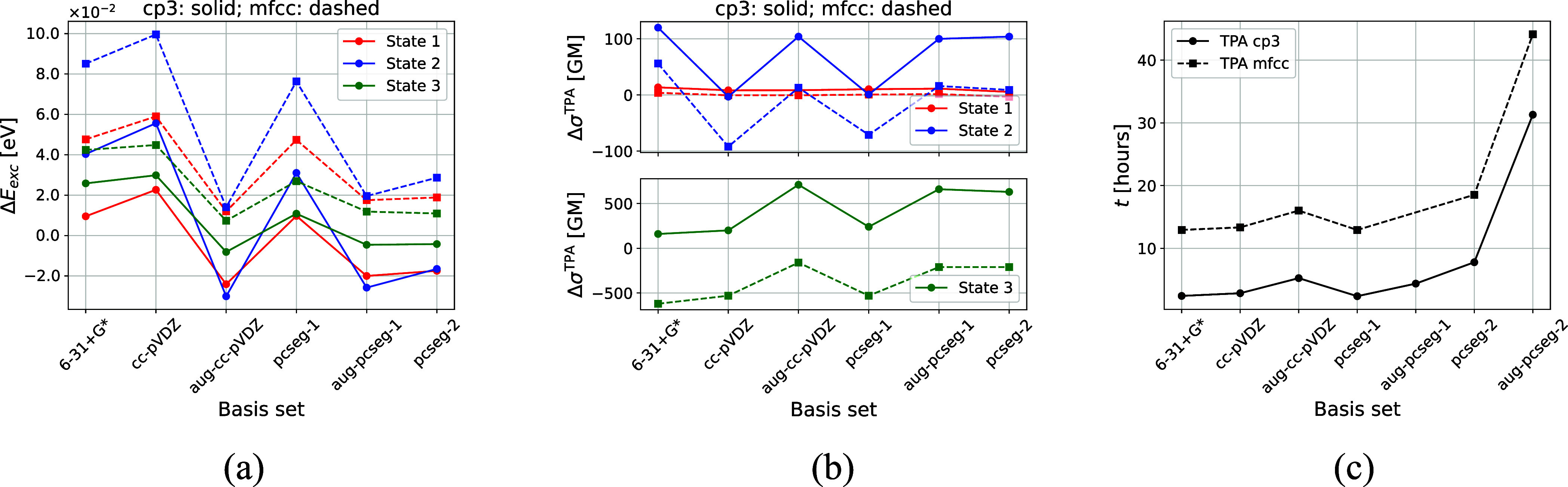
Basis-set dependence of excitation energies (a) and TPA cross sections (b) of a random snapshot from the QM/MM-MD trajectory for the ***mfcc*** and ***cp3*** approaches investigated. Reference values are based on the ***mfcc*** approach and aug-pcseg-2 basis set. All values calculated at PE-TD-DFT level of theory using CAM-B3LYP and *FMM* (θ = 0.5, *p* = 5), ESPF (*p* = 0, *R*_exact_ = 10), and pseudopotentials on all MM atoms belonging to fragments (excluding lipids) that have at least one atom within 6 Å from any atom of the RSB chromophore. Timings of the ***mfcc*** and ***cp3*** approaches are given in plot (c) (more information in the main text).

### 4.3 Spectra

A key consideration in generating the absorption spectra from an MD trajectory is determining the optimal number of snapshots and their spacing to ensure statistically uncorrelated data. If frames are sampled too densely, adjacent data remain correlated, thus, adding snapshots repeats the same information. To choose an appropriate spacing, we analyzed the autocorrelation function of the TPA cross-section defined as

6 $$C\left(t\right)=\frac{1}{n\sigma^{2}}\overset{n-k}{\underset{t=1}{\sum }}\left(\sigma_{t}^{\mathrm{TPA}}-\mu \right)\left(\sigma_{t+k}^{\mathrm{TPA}}-\mu \right)$$

where *n* is the total number of snapshots, σ^2^ and μ are the variance and mean of the TPA cross sections $\sigma_{t}^{\mathrm{TPA}}$ , respectively, and the summation runs over all pairs of snapshots separated by *k*, which is the number of time steps separating the pair of snapshots being compared (here *k* = 1). The TPA cross sections were calculated with PE-TD-DFT using CAM-B3LYP, the 6-31+G* basis set, and the ***cp3*** embedding potential. This analysis was performed on the last 5 ps of the QM/MM-MD trajectory, where 1000 snapshots were collected at 5 fs intervals. Figure 9, shows the autocorrelation function fitted to an exponential decay model *C*(*t*) = *A* exp­(−*t*/τ), where *A* is the amplitude at *t* = 0 and τ is the characteristic decay time. The rapid decay of *C*(*t*) indicates that uncorrelated configurations with respect to their TPA cross-section can be found every 200 fs.

**Figure 9:**
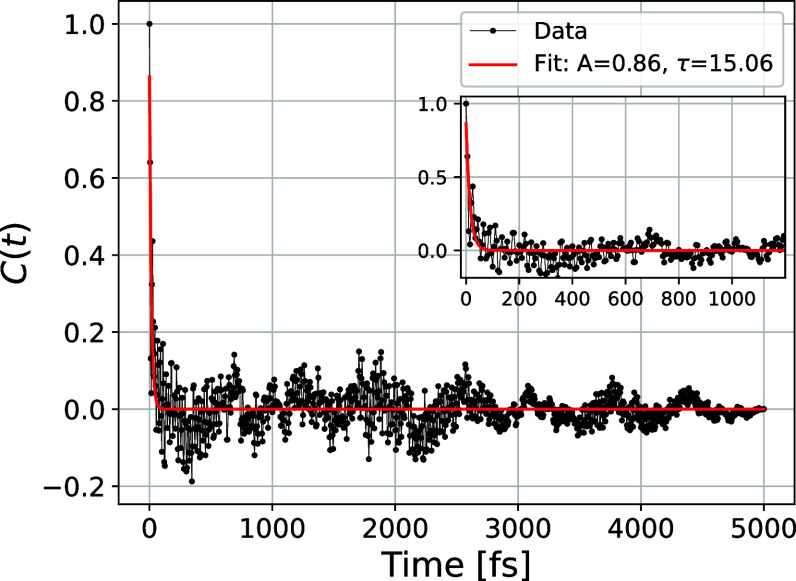
Autocorrelation function of the TPA cross-section associated with the lowest transition. It is computed using the last 5 ps of the QM/MM-MD trajectory. Black dots: data. Red line: exponential fit *C*(*t*) = *A* exp­(−*t*/τ) giving *A* = 0.86 and τ = 15 fs. The inset highlights the rapid decay, which suggests that sampling the trajectory at 200 fs intervals yields uncorrelated configurations with respect to their TPA cross-section.

Having established from *C*(*t*) that a 200 fs spacing yields effectively uncorrelated configurations, we next examined how many such snapshots are needed for converged and adequate spectra. We therefore down-sampled the same 40 ps production trajectory at spacings of 200, 400, 800, and 1600 fs, which correspond to *N* = 200, 100, 50, and 25 independent snapshots, respectively. Peak positions and maxima are essentially unchanged across *N* (Figure S2), while increasing *N* mainly smooths the spectra and produces more nearly Gaussian distributions of *E*_exc_, *f*_osc_, and σ^2PA^ (Figure S3). As a practical choice, we consider *N* = 100 a reasonable minimum and adopt *N* = 200 hereafter to be conservative.

For each snapshot, we generate a ***cp3*** embedding potential and conduct a PE-TD-DFT calculation with the aug-pcseg-1 basis set. For each snapshot, this calculation is performed twice, where the core quantum region is either the LYR-472 (QM-sampled) or LYR-225 (MM-sampled) moieties, as highlighted in Figure 10.

**Figure 10:**
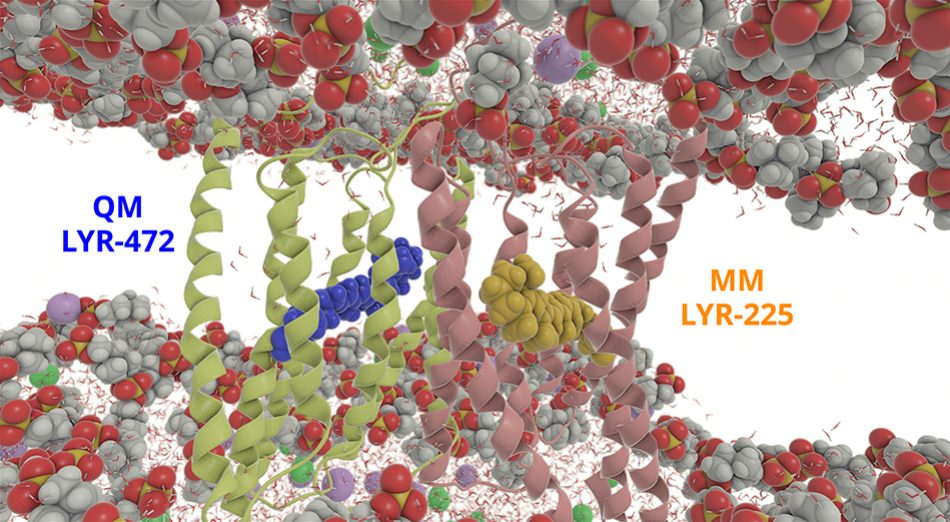
Zoomed view of the ChR2 system, highlighting the two RSB moieties sampled simultaneously along the QM/MM-MD trajectory: LYR-472 (QM-sampled) and LYR-225 (MM-sampled). POPC lipid tails are omitted for clarity, showing only the heads.

The OPA, TPA and 3PA spectra are calculated by reading the excitation energies, oscillator strengths, and one-, two- and three-photon transition strengths from the Dalton outputs, including up to the third excited state (*i* = 3 in eqs 2 and 3), and applying Lorentzian broadening with γ_*i*_ = 0.1 with the help of the Dalton Project Python package.^95^ Figure 11 shows the theoretical and experimental OPA spectra. To facilitate comparison, the experimental intensities are multiplied by a scaling factor so that the maximum of the experimental lowest-energy band matches the one from the QM-sampled moiety. Peak energies and deviations are listed in Table 1. The absorption maximum for the QM-sampled moiety closely aligns with the experimental result, showing a deviation of 0.17 eV for the first excited state. In contrast, the MM-sampled moiety exhibits a much larger deviation of 1.02 eV. The experiment shows an intense peak at 4.5 eV,^5^ which is not reproduced theoretically, potentially due to tryptophan residues in ChR2, which contains up to 10 tryptophans in total absorbing in this region.^104^

**Table 1:** Energies in eV for the One-Photon Absorption Spectra Shown in Figure 1

	Exp.^a^	QM LYR-472		MM LYR-225	
Excited state no.	*E*	*E*	Δ*E* ^b^	*E*	Δ*E* ^b^
1	2.64	2.81	0.17	3.66	1.02
2	–	4.13	–	5.17	–

a Experiment from ref 5.

b Difference with respect to the experiment.

**Figure 11:**
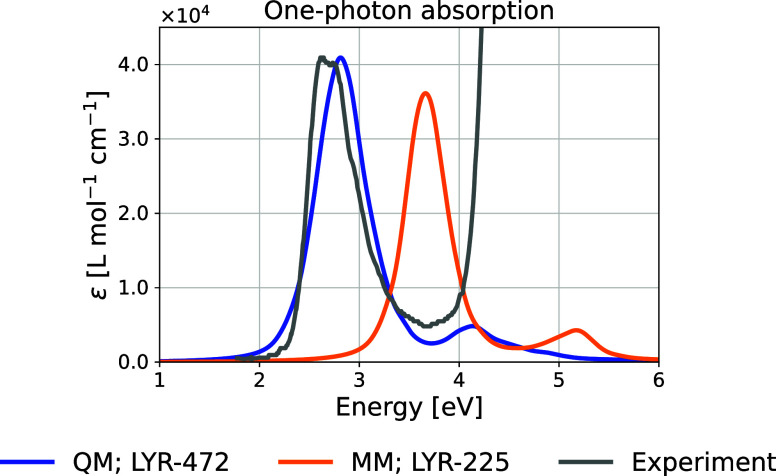
Theoretical and experimental^5^ one-photon absorption spectra. The theoretical spectra show the LYR-472 (QM-sampled) and LYR-225 (MM-sampled) moieties. The *x*-axis refers to photon energies.

The TPA spectra for the QM- and MM-sampled moieties are shown in Figure 12. To the best of our knowledge, no experimental TPA spectra have been reported for this system for direct comparison. The differences in excitation energies between both moieties are summarized in Table 2, showing deviations of 0.38 and 0.34 eV for the first and second excited states, respectively. As for the intensities, the difference in the peak intensity of the first excited state for both moieties is minor (just 9.22 GM), while the intensity of the second excited state for the QM-sampled moiety is notably higher than that of the MM-sampled moiety (1718.95 GM difference).

**Table 2:** Energies in eV and Intensities in GM respectively, for the Two-Photon Absorption (TPA) Spectra Shown in Figure 12

	QM LYR-472	MM LYR-225	
Excited state no.	*E*	*E*	Δ*E* ^a^
1	1.45	1.83	0.38
2	2.22	2.56	0.34

Excited state no.	*I*	*I*	Δ*I* ^a^
1	32.91	23.69	9.22
2	1825.42	106.47	1718.95

a Energy and intensity difference between MM- and QM-sampled moieties.

**Figure 12:**
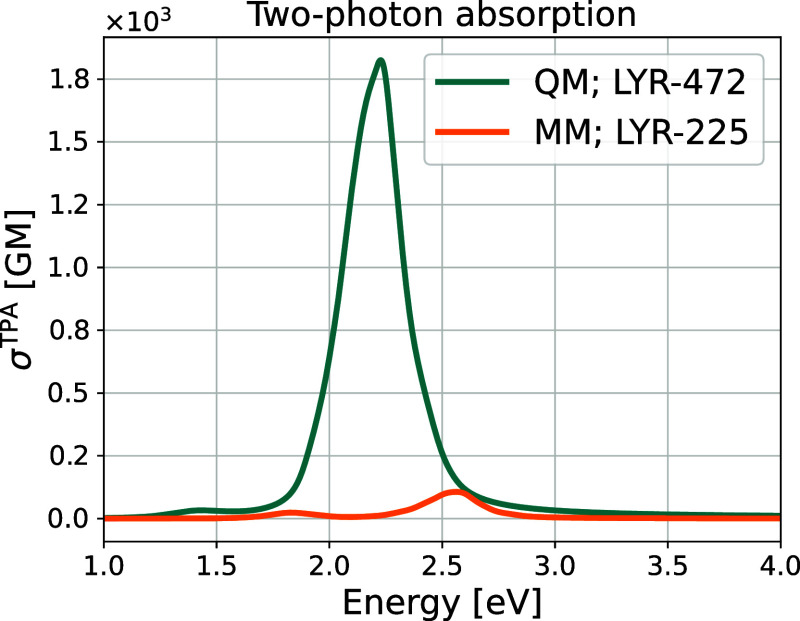
Theoretical two-photon absorption spectra for the QM-sampled (LYR-472) and MM-sampled (LYR-225) moieties.

Figure 13 compares the OPA and TPA spectra with and without considering the environment. While the line shape and intensities differ significantly, the position of the peaks show less but noticeable deviations. For the OPA spectra, the first and second excited states exhibit differences of 0.37 and 0.62 eV, respectively. As for the intensities, the first and second excited states differ by 59063 and 10577 L mol^–1^ cm^–1^, respectively. In the case of the TPA spectra, the deviations are smaller, with differences of 0.21 and 0.06 eV for the first and second excited states, respectively. As for the intensities, the first and second excited states differ by 233 and 967 GM, respectively.

**Figure 13:**
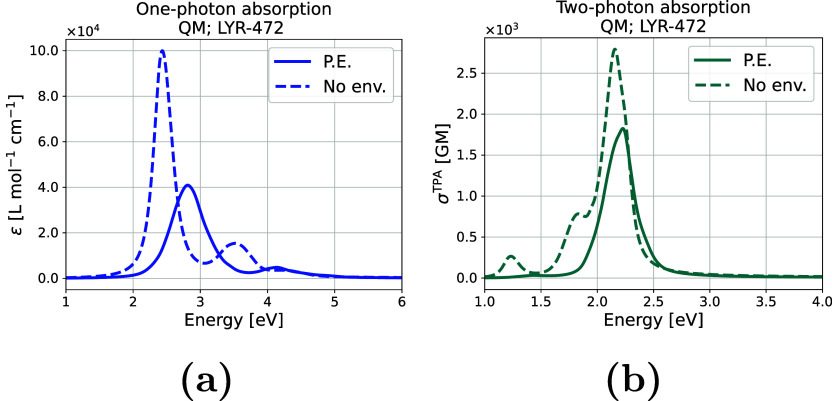
Comparison of one-photon absorption spectra (a) and two-photon absorption spectra (b) for the QM-sampled moiety (LYR-472), with and without polarizable embedding, for the 200 snapshots analyzed.

**Figure 14:**
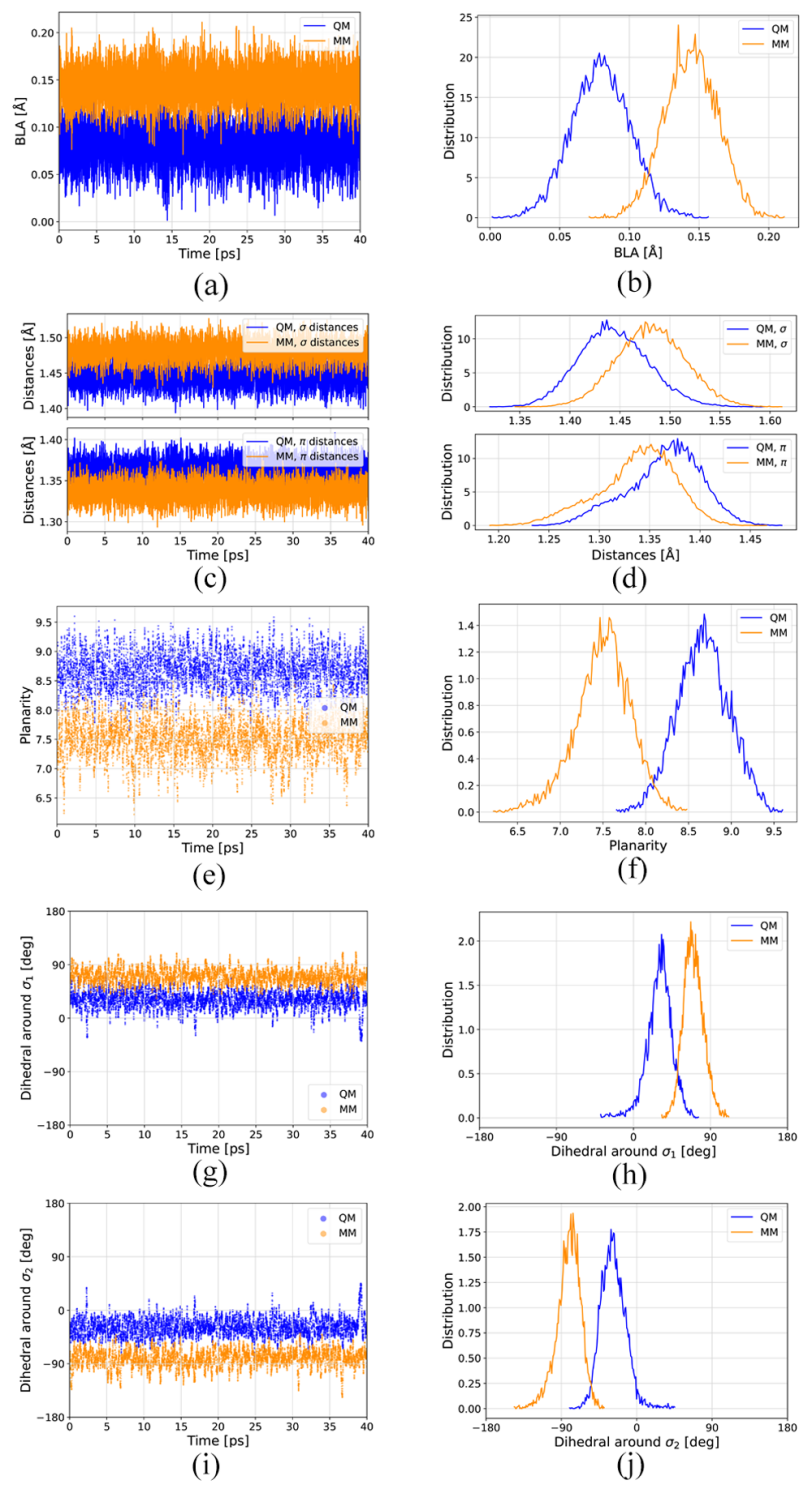
Timeline (left panel) and distributions (right panel) of different structural parameters for the QM-sampled (LYR-472) and MM-sampled (LYR-225) moieties: (a,b) bond-length alternation (BLA); (c,d) single- and double-bond distances; (e,f) planarity; (g,h) dihedrals around σ_1_ bonds; and (i,j) dihedrals around σ_2_ bonds (which are those dihedrals that are mainly responsible for the different planarity).

The combined OPA, TPA, and 3PA spectra are shown in Figure 15. Table 3 summarizes the energy values corresponding to the peak intensities. The first and second excited states appear at 2.81 and 4.13 eV in OPA, which are approximately halved in TPA to 1.45 and 2.22 eV, respectively.

**Table 3:** Energies in eV for the One-, Two- and Three-Photon Absorption Spectra for the QM-Sampled Moiety (LYR-472), Data That Corresponds to Fig. 15

Excited state no.	OPA	TPA	3PA
1	2.81	1.45	–
2	4.13	2.22	1.51

**Figure 15:**
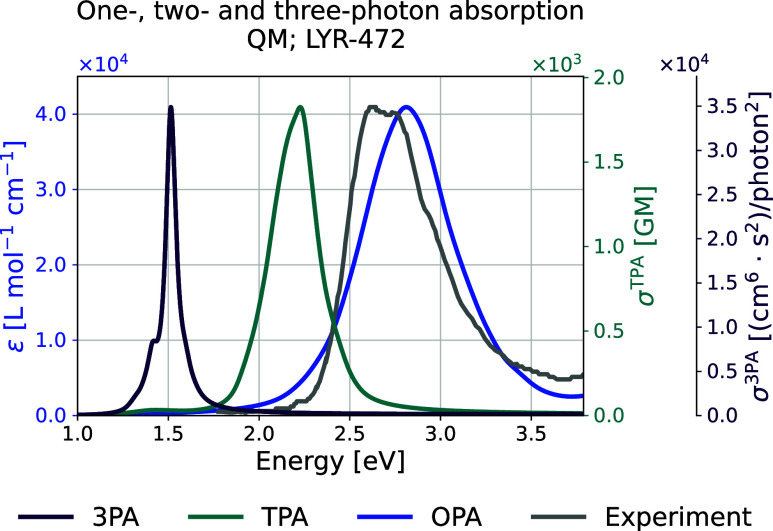
Theoretical one-, two-, and three-photon absorption spectra for the QM-sampled moiety (LYR-472).

For 3PA, the peak primarily corresponds to the second excited state, as this transition exhibits the highest transition strength across all snapshots, dominating the 3PA spectrum.

### 4.4 Structural Parameters

To understand the structural differences that account for the observed variations in the spectra, we analyzed the bond-length alternation (BLA) and planarity of the QM- and MM-sampled moieties. The BLA is the average difference in bond lengths between the single and double C–C bonds in the backbone:

7 $$\mathrm{BLA}=\frac{1}{5}\left[\overset{5}{\underset{i=1}{\sum }}\sigma_{i}-\overset{5}{\underset{i=1}{\sum }}\pi_{i}\right]$$

where σ_*i*_ and π_*i*_ are the single- and double-bond lengths, respectively (see Figure 3b). Figure 14a,b shows the time evolution and distribution of the BLA along the QM/MM-MD trajectory as obtained with the scripting functionality in VIAMD.^105^ The BLA is consistently lower for the QM-sampled moiety.

The timeline and distribution of the single- and double-bond distances are shown in Figure 14c and d, respectively. C–C single and double-bond distances are overestimated and underestimated, respectively, in the MM-sampled moiety, leading to a higher BLA.

We have analyzed the dihedral angles around the σ_*i*_ and π_*i*_ bond distances for *i* = 1, 2, 3, 4 and 5, in total, 10 dihedral angles. The planarity *P* can then be defined as^106^

8 $$P=\overset{10}{\underset{i=1}{\sum }}p_{i}=\overset{10}{\underset{i=1}{\sum }}\frac{\left|\left|\theta_{i}\right|-90\right|}{90}$$

For each bond, a dihedral angle θ_*i*_ = ±90° indicates that the two groups around the *i*-th single or double bond are completely out of plane with each other (*p*_*i*_ = 0), while θ_*i*_ = {0°,180°} indicates that they are completely planar (*p*_*i*_ = 1).

For each dihedral, a value of *p*_*i*_ between 0 and 1 is added, so that if all dihedrals were planar, the planarity of the molecule would be *P* = 10, while if all dihedrals were completely out of plane, then *P* = 0. A planarity value around 8.7 is observed for the QM-sampled moiety, while this value decreases to 7.5 for the MM-sampled moiety (see timeline and distributions in Figure 14e,f, respectively). The higher planarity observed for the QM-sampled moiety indicates that this structure has a higher degree of conjugation. We have evaluated all ten dihedrals, and those that cause this difference in planarity are mainly those around σ_1_ (Figure 14h,g) and σ_2_ (Figure 14j,g). The rest of the dihedrals (σ_*i*_ and π_*i*_ where *i* = 3, 4 and 5) are very similar in the MM- and QM-sampled moieties (see Figure S4).

We have evaluated the correlation between the spectroscopic properties and structural parameters (BLA and planarity). The QM-sampled moiety exhibits systematically smaller BLA and higher planarity, the latter mainly due to dihedrals around σ_1_ and σ_2_. The lower the BLA, the more equidistant the single and double C–C bonds are, which is connected to higher conjugation and hence lower first excitation energy, which is what we see across snapshots (Figure 16a). Similarly, more planar geometries come with lower first excitation energies (Figure 16b). These correlations are consistent with the redshift observed in the OPA spectrum for the QM-sampled moiety compared to the MM-sampled moiety (Figure 11).

**Figure 16:**
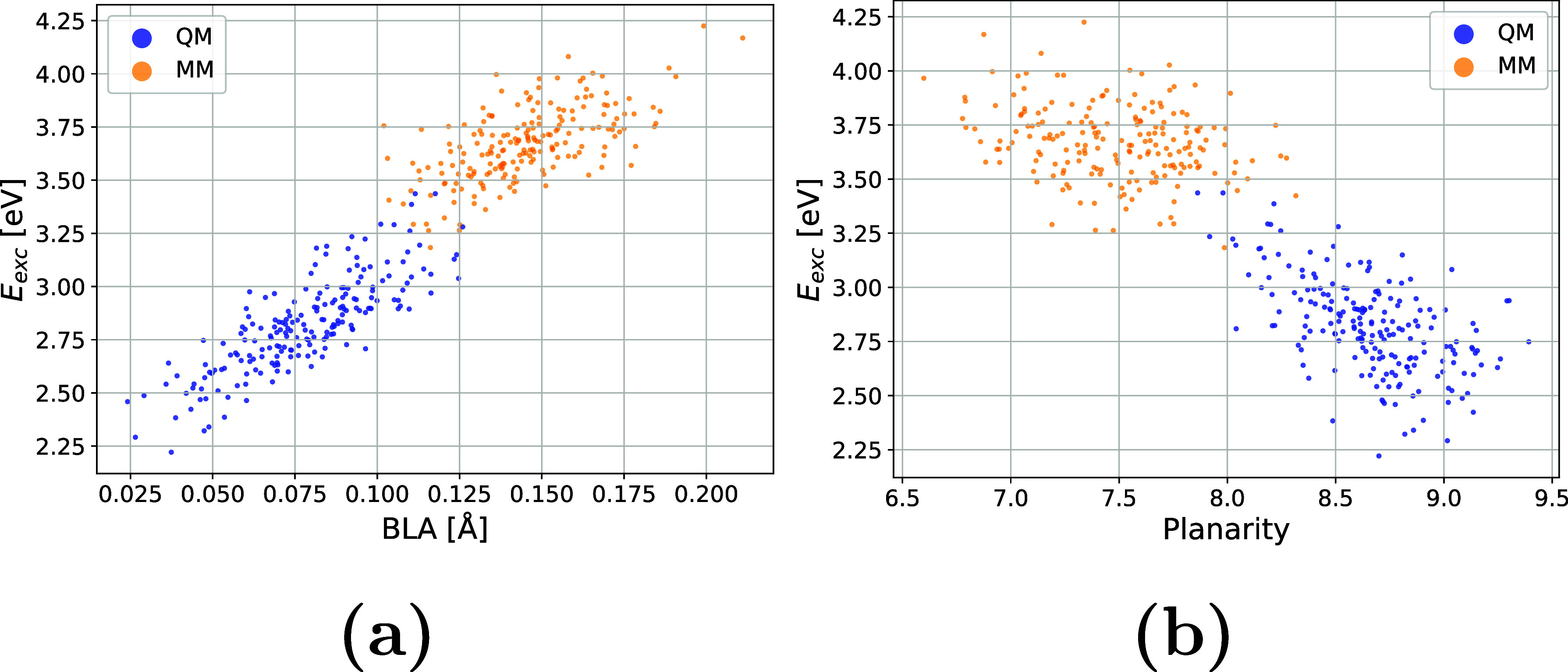
First excitation energies against (a) BLA and (b) planarity for the QM-sampled (LYR-472) and MM- sampled (LYR-225) moieties.

## 5 Conclusion and Outlook

We have presented a fully atomistic multiscale methodology for generating the MPA spectra of ChR2, which can be easily extended to other light-sensitive membrane-protein systems (e.g., other opsins, retinal proteins, fluorescent proteins, etc.). This approach combines classical MD with QM/MM-MD sampling, followed by fragment-based PE-TD-DFT calculations to obtain accurate spectra using embedding potentials that describe the protein–lipid-solvent environment.

The entire transmembrane protein system, including the lipid bilayer, water, and ions, was explicitly considered throughout the workflow. A key aspect of this study is the use of QM/MM-MD to extend configurational sampling beyond classical MM-MD. We determined the optimal spacing between snapshots to ensure uncorrelated structures with respect to spectroscopic properties. In the QM/MM-MD, the two RSB moieties were described by QM and MM, respectively. This allowed us to analyze the effect of MM versus QM/MM-MD sampling on the spectra. The QM-sampled OPA absorption maximum agrees closely with the experiment, achieving an error of only 0.17 eV for the first peak, whereas the MM-sampled moiety is substantially blue-shifted. Although an identical PE-TD-DFT workflow was used for both ensembles, the different spectra are attributable to the underlying force field used for the MM-sampled moiety.

We have linked the differences in the spectra between the QM- and the MM-sampled moieties to structural parameters, i.e., BLA and planarity. We show that the lower BLA and higher planarity (that is, a higher degree of conjugation) observed in the QM-sampled moiety correlate with a lower first electronically excited state energy, hence explaining the redshift observed in the QM-sampled OPA spectra compared to the MM-sampled moiety.

For the first time, we have also generated the theoretical TPA and 3PA spectra for ChR2. Furthermore, we have quantified the effect of environments on the spectra with respect to not including the environment at all. Our results demonstrate the critical role of environment effects in accurately modeling the spectroscopic behavior of complex biomolecular systems. We have documented the methodology at each stage of the workflow and provided a detailed account of the fragment-based description of the environment, with particular attention to the approximations used to capture the contributions from the environment.

In summary, the methodology presented in this paper provides a solid basis for MPA spectra predictions that may aid the experimental elucidation of competing photocycle models in ChR2 and engineered rhodopsin variants, helping to advance a bottom-up, structure-guided development of improved optogenetic tools. From a computational perspective, some steps of this workflow, such as snapshot selection, environment fragmentation, embedding-parameter generation, and PE-TD-DFT execution can be automated even further within Python–based quantum–classical interoperable platforms,^107^ enabling protocols that are even more reproducible and fully end-to-end for complex light-sensitive membrane-protein systems.

## Supplementary Material



## Data Availability

The relevant scripts, along with all workflow instructions and input files necessary to reproduce the results presented in this paper, are available in ref 102. The relevant molecular dynamics trajectories can be found in ref 103.

## Notes

Additional system preparation and molecular dynamics computational details; additional tables regarding approximations in polarizable embedding (approximations on the MM fields, QM/MM interactions, and the impact that placing pseudopotentials on MM atoms has on the spectroscopic properties of the RSB moiety); one- and two-photon absorption spectra of QM- and MM-sampled moieties with respect to the number of frames, as well as the normal distribution of spectroscopic properties with respect to the number of frames; additional figures showing the distributions and timelines of the remaining dihedrals in RSB, the RMSD, and the H–GLU_123_ distance; all spectroscopic properties against BLA and planarity; shape space of the QM- and MM-sampled moieties over a 1 μs MM-MD trajectory and the 45 ps QM/MM-MD trajectory (PDF)
