# Supplementary material for: Multiphoton Absorption Spectra of Channelrhodopsin‑2 via Multiscale Simulation Methods
Source: J Chem Theory Comput. 2026 Jan 6;22(2):1133–48. doi: 10.1021/acs.jctc.5c01719 (PMC12854767; doi:10.1021/acs.jctc.5c01719)
Supplement: Supplementary file 1 [file ct5c01719_si_001.pdf]

Supporting Information: Multiphoton Absorption Spectra of Channelrhodopsin-2 via Multiscale Simulation Methods

David Carrasco-Busturia,[*,†] Mathieu Linares,[‡] Patrick Norman,[†] and Jógvan Magnus Haugaard Olsen[*,¶]

†*Division of Theoretical Chemistry and Biology, School of Engineering Sciences in Chemistry, Biotechnology and Health, KTH Royal Institute of Technology, SE-100 44 Stockholm, Sweden*

‡*PDC Center for High Performance Computing, KTH Royal Institute of Technology, SE-100 44, Stockholm, Sweden*

¶*DTU Chemistry, Technical University of Denmark, DK-2800 Kgs. Lyngby, Denmark*

E-mail: davidcdb@kth.se; jmho@kemi.dtu.dk

1 System Preparation

Table S1: Summary of the restraints applied in each of the six MM-equlibration runs

Run	Ensemble	δt (fs)	Time (ps)	k_z^{P} (kJ mol^{-1} nm^{-2})	θ_0^{GL} (deg)	$\Delta\theta^{\mathrm{GL}}$ (deg)	k_{dihr}^{GL} (kJ mol^{-1} nm^{-2})	θ_0^{OL} (deg)	$\Delta\theta^{\mathrm{OL}}$ (deg)	k_{dihr}^{OL} (kJ mol^{-1} nm^{-2})	k^{BB} (mol^{-1} nm^{-2})	k^{SC} (mol^{-1} nm^{-2})
1	NVT	1	125	1000	120	2.5	1000	0	0	1000	4000	2000
2	NVT	1	125	400	120	2.5	400	0	0	400	2000	1000
3	NPT	1	125	400	120	2.5	200	0	0	200	1000	500
4	NPT	2	500	200	120	2.5	200	0	0	200	500	200
5	NPT	2	500	40	120	2.5	100	0	0	100	200	50
6	NPT	2	500	0	120	2.5	0	0	0	0	50	0

Several potentials were used for imposing restraints on the motion of the POPC bilayer during the energy minimization step: i) a position restraint on the phosphor atoms of the phosphate groups was applied in the z direction with a force constant of $k_z^{\mathrm{P}} = 1000$ kJ·mol^{-1}·nm^{-2} to keep the thickness of the bilayer, ii) the dihedral angle C1-C3-C2-O21 corresponding to the glycerol (GL) head is restrained to a reference angle $\theta_0^{\mathrm{GL}} = 120°$, $\Delta\theta^{\mathrm{GL}} = 2.5°$ with a force constant of $k_{dihr}^{\mathrm{GL}} = 1000$ kJ mol^{-1} nm^{-2} to keep the stereoisomer, and iii) the dihedral angle C18-C19-C110-C111 corresponding to the oleoyl chain (OL) was restrained to $\theta_0^{\mathrm{OL}} = \Delta\theta^{\mathrm{OL}} = 0°$ with a force constant of $k_{dihr}^{\mathrm{OL}} = 1000$ kJ mol^{-1} nm^{-2} to keep the *cis*-isomer. Atom labels P31, C1–C3, O21, C18, C19, C110, and C111 follow the POPC atom types shown in Fig. S1. Similarly, several position restraints were activated in both chains of the protein: i) a position restraint in the backbone (BB) atoms was applied with a force constant of $k^{BB} = k_x = k_y = k_z = 4000$ mol^{-1} nm^{-2} and ii) for the side-chain (SC) atoms with a force constant of $k^{SC} = k_x = k_y = k_z = 2000$ mol^{-1} nm^{-2}. After the energy minimization step, six equilibration steps were performed (Table S1). The first step runs with the same restraints as the energy minimization step. From the second equilibration step onwards, restraints were progressively removed. The first two are NVT runs, where the velocity-rescaling thermostat is employed,[S1] with 1 ps time constant and temperature set to 303.15 K, which is above the phase transition temperature of the POPC bilayer.[S2] Three different groups are coupled separately due to the different rates of diffusion of the three phases: *i*) the protein *ii*) the lipid membrane *iii*) water and ions. For the four remaining NPT equilibration runs, a semi-isotropic pressure coupling (isotropic in the x and y direction,

but different in the z direction, to allow the lipid bilayer to deform in the xy plane independently of the z-axis) is applied through the Berendsen barostat[S3] with a coupling constant of 5 ps and a reference pressure of 1 bar and $4.5 \cdot 10^{-5}$ bar^{-1} compressibility. A velocity Verlet integrator[S4] with a timestep of 1 fs for the first two equilibration runs and 2 fs for the rest of the runs was used. In order to avoid membrane and water systems from moving laterally and possibly drifting in opposite directions, the motions of the center of mass of the lipid bilayer and the solvent were reset separately through the `comm-grps` keyword.

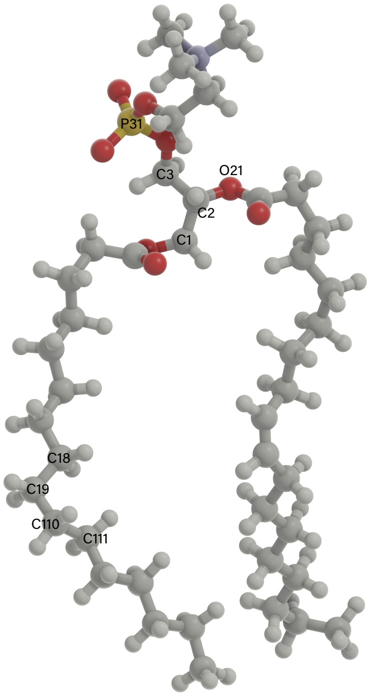

Figure S1: Atom labels (P31, C1–C3, O21, C18, C19, C110, and C111) in POPC

2 Approximations in PE

Table S2: Approximations on the MM fields with the Fast Multipole Method (*FMM*). Parameters θ and p are, respectively, the multipole acceptance criteria, and the multipole expansion order All values at PE/CAM-B3LYP/6-31+G* level of theory and using the ***cp3*** approach for the calculation of the embedding potential.

States	*FMM* $\theta = 0.4$, $p = 6$	*FMM* $\theta = 0.5$, $p = 5$
	E_{exc}(eV)	
1	2.6265	2.6266
2	4.0709	4.0709
3	4.3414	4.3416
	f_{osc}	
1	1.0130	1.0120
2	0.0335	0.0333
3	0.0611	0.0610
	σ^{TPA} (GM)	
1	83.2	82.9
2	827.	826.
3	5510.	5490.
	Time, TPA (h)	
	13.4	12.4

Table S3: Approximations on the QM/MM interactions with the *single-center* and *ESPF* methods using FMM (with $\theta = 0.5$ and $p = 5$). All values at PE/CAM-B3LYP/6-31+G* level of theory, and using the ***cp3*** approach for the calculation of the embedding potential.

States	*FMM*, *Single-center*, $p = 8$, $R_{\text{exact}} = 65$	*FMM*, *Single-center*, $p = 8$, $R_{\text{exact}} = 40$	*FMM*, *ESPF*, $p = 0$, $R_{\text{exact}} = 10$
		E_{exc}(eV)	
1	2.6265	2.6265	2.6264
2	4.0709	4.0709	4.0703
3	4.3414	4.3414	4.3413
		f_{osc}	
1	1.0128	1.0128	1.0126
2	0.0335	0.0335	0.0334
3	0.0611	0.0611	0.0612
		σ^{TPA}	
1	83.2	83.2	83.2
2	827.	827.	829.
3	5510.	5510.	5500.
		Time, TPA (h)	
	4.6	4.2	3.3

Table S4: The impact of placing pseudopotentials (PPs) on the computed excitation energies, oscillator strengths, and TPA cross-sections. PPs are placed on all atoms belonging to fragments (excluding lipids) that have at least one atom within 6 Å from any atom of the RSB chromophore. The hydrogen link atom is described using the STO-3G basis set. All values at PE/CAM-B3LYP/aug-pcseg-1 level of theory, and using the ***cp3*** approach for the calculation of the embedding potential. Approximations on the MM fields and QM/MM interactions are *FMM* ($\theta = 0.5$, $p = 5$), and *ESPF* ($p = 0$, $R_{exact} = 10$) respectively.

cp3 approach	State 1	State 2	State 3
		E_{exc} (eV)	
Without PPs	2.6132855	4.0425968	4.3257549
	$94 \rightarrow 95$	$94 \rightarrow 96$	$93 \rightarrow 95$
With PPs (and H-link atom with STO-3G)	2.6541096	4.1144944	4.3461336
	$94 \rightarrow 95$	$94 \rightarrow 96$	$93 \rightarrow 95$
	$\Delta E = 0.04$	$\Delta E = 0.07$	$\Delta E = 0.02$
		f_{osc}	
Without PPs	1.0108524	0.0350	0.0565
With PPs (and H-link atom with STO-3G)	1.0245079	0.0378	0.0476
	$\Delta f = 0.01$	$\Delta f = 0.003$	$\Delta f = 0.009$
		σ^{TPA}	
Without PPs	79.3	835.	5750.
With PPs (and H-link atom with STO-3G)	62.7	776.	5190.
	$\Delta\sigma^{\text{TPA}} = 16.6$	$\Delta\sigma^{\text{TPA}} = 59$	$\Delta\sigma^{\text{TPA}} = 560$

Table S5: The impact of placing pseudopotentials (PPs) on the computed excitation energies, oscillator strengths, and TPA cross-sections. PPs are placed on all atoms belonging to fragments (excluding lipids) that have at least one atom within 6 Å from any atom of the RSB chromophore. The hydrogen link atom is described using the STO-3G basis set. All values at PE/CAM-B3LYP/aug-pcseg-1 level of theory, and using the ***mfcc*** approach for the calculation of the embedding potential. Approximations on the MM fields and QM/MM interactions are *FMM* ($\theta = 0.5$, $p = 5$), and *ESPF* ($p = 0$, $R_{exact} = 10$), respectively [1]

mfcc approach	State 1	State 2	State 3
	E_{exc} (eV)		
Without PPs	2.0800132	2.6382855	2.7622638
	$94 \rightarrow 95$	$94 \rightarrow 100$	$94 \rightarrow 97$
With PPs (and H-link atom with STO-3G)	2.6916428	4.1598389	4.3625780
	$94 \rightarrow 95$	$94 \rightarrow 96$	$93 \rightarrow 95$
	$\Delta E = 0.61$	$\Delta E = 1.5$	$\Delta E = 1.6$
	f_{osc}		
Without PPs	0.00487748	0.96710367	0.00270386
With PPs (and H-link atom with STO-3G)	0.99878982	0.04512540	0.03917137
	$\Delta f = 0.994$	$\Delta f = 0.92$	$\Delta f = 0.036$
	σ^{TPA}		
Without PPs	2.86	74.2	0.553
With PPs (and H-link atom with STO-3G)	53.	692.	4320.
	$\Delta\sigma^{\text{TPA}} = 50.1$	$\Delta\sigma^{\text{TPA}} = 617.8$	$\Delta\sigma^{\text{TPA}} = 4319.4$

[1] Such a low oscillator strength (0.00487748) as well as low excitation energy (2.08 eV) reported in State 1, without PPs, indicates that this is a spurious excited state that most likely appears because of electron spill-out.

3 Spectra

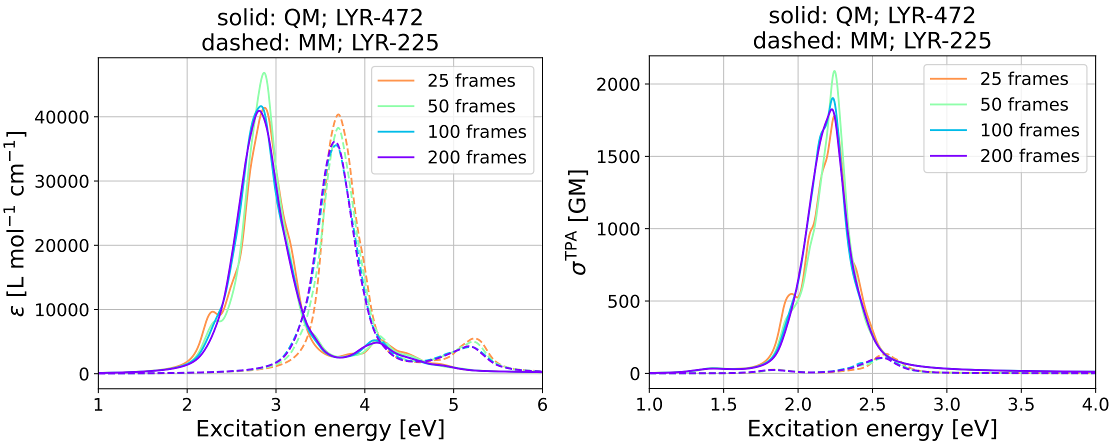

(a) (b)

Figure S2: One- and two-photon absorption spectra for the QM- and MM-sampled moieties with respect to the number of snapshots (25, 50, 100, and 200).

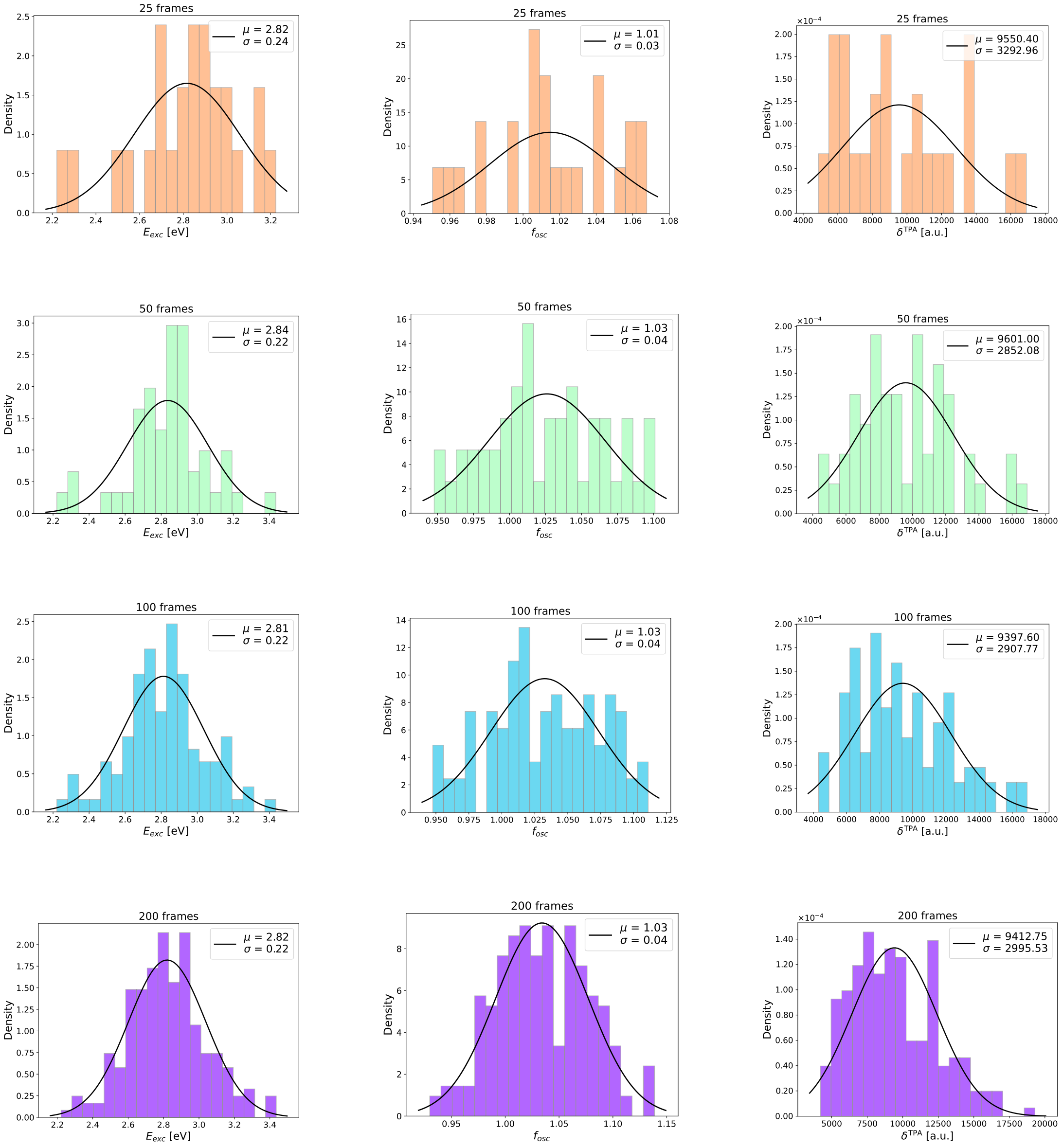

Figure S3: Spectroscopic properties (first excitation energy, first oscillator strength, and first two-photon electronic transition strength) over 25, 50, 100 and 200 snapshots for the QM-sampled moiety (QM; LYR-472). In black, the normal distribution. The mean value and standard deviation are shown in the legend of each figure.

4 Structural parameters

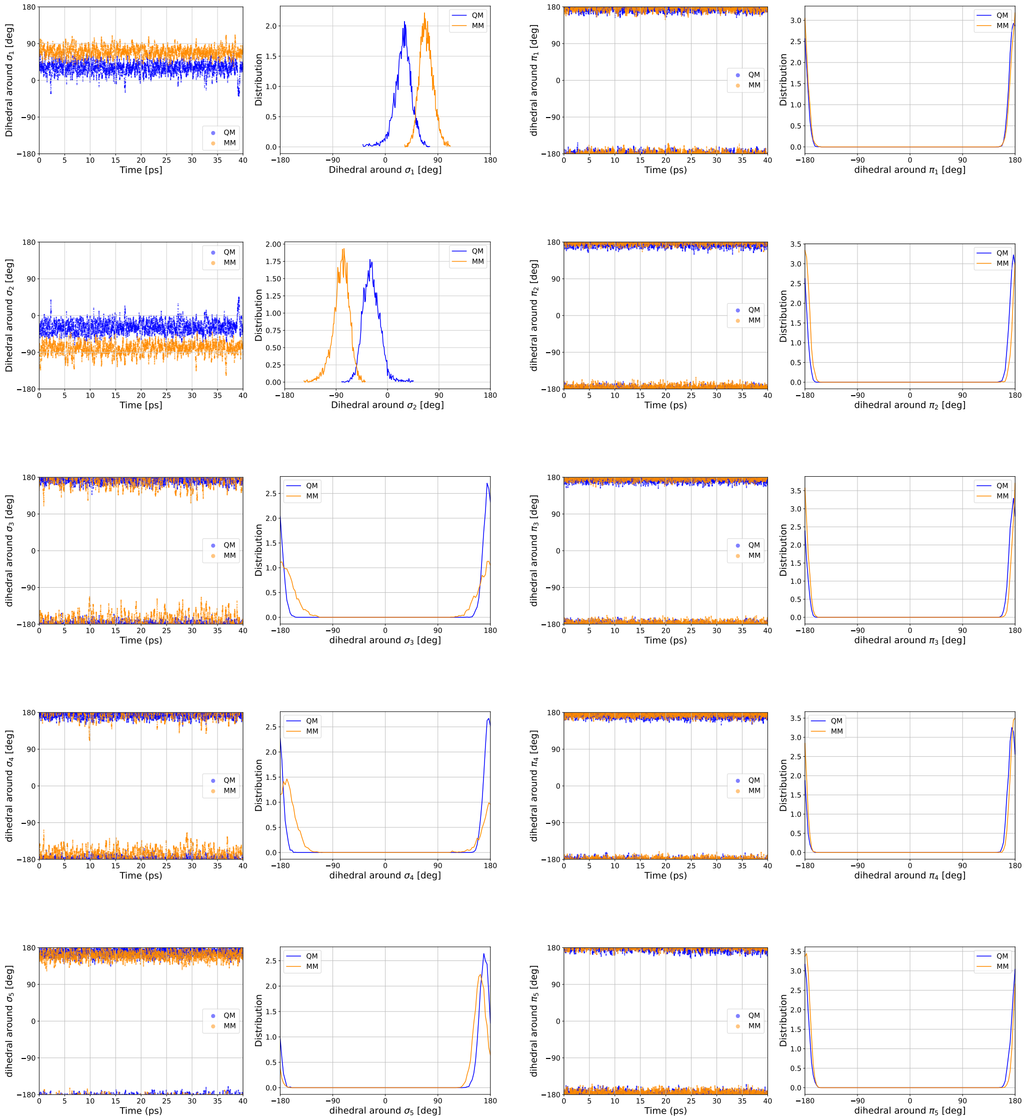

Figure S4: Distribution and timelines along the 40 ps QM/MM-MD trajectory for the dihedrals around σ_i and π_i where $i = 1, 2, 3, 4, 5$.

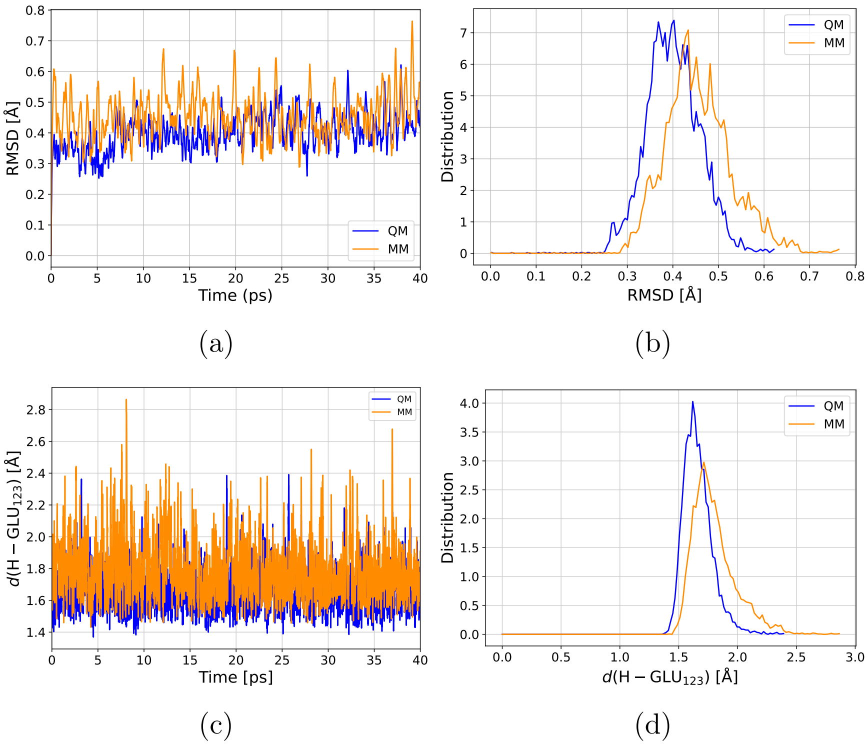

Figure S5: RMSD, and the distance H—GLU_{123} shown in Fig. 3a in the main text. Both timeline and distributions are shown.

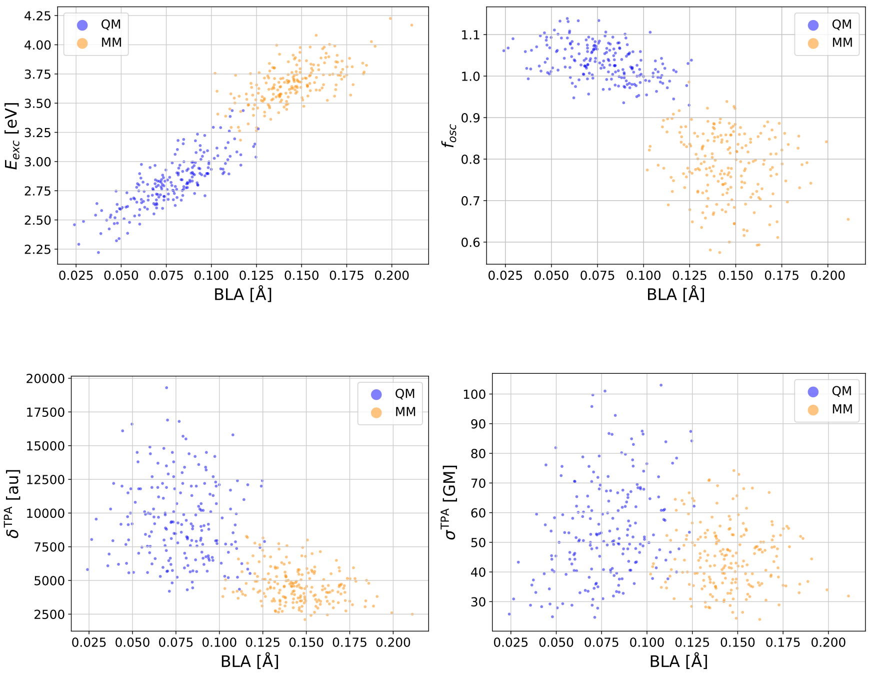

Figure S6: Spectroscopic properties for the first excitation (oscillator strengths, transition probability and two-photon absorption cross-section) against BLA

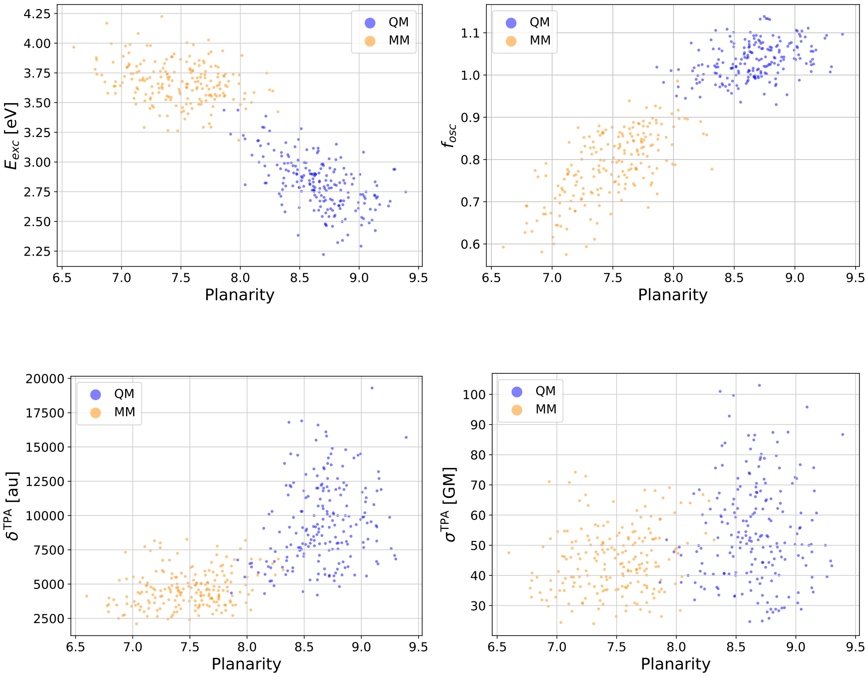

Figure S7: Spectroscopic properties for the first excitation (oscillator strengths, transition probability and two-photon absorption cross-section) against planarity

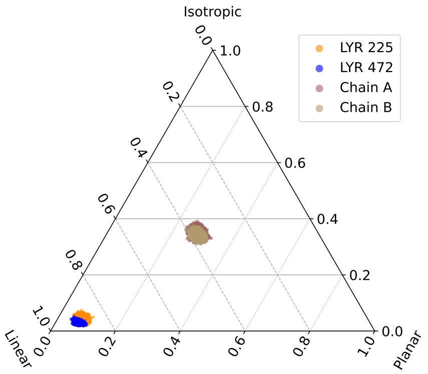

(a)

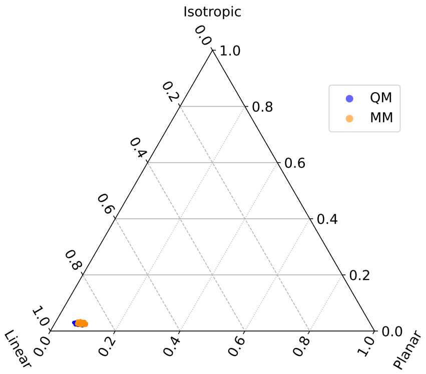

(b)

Figure S8: Shape space over the 1 microsecond MM MD (S8a) and the 45 ps QM/MM MD trajectory (S8b).

References

(S1) Bussi, G.; Donadio, D.; Parrinello, M. Canonical sampling through velocity rescaling. *The Journal of Chemical Physics* **2007**, *126*, 014101.

(S2) Tieleman, D. P.; Forrest, L. R.; Sansom, M. S. P.; Berendsen, H. J. C. Lipid Properties and the Orientation of Aromatic Residues in OmpF, Influenza M2, and Alamethicin Systems: Molecular Dynamics Simulations. *Biochemistry* **1998**, *37*, 17554–17561, PMID: 9860871.

(S3) Berendsen, H. J. C.; Postma, J. P. M.; van Gunsteren, W. F.; DiNola, A.; Haak, J. R. Molecular dynamics with coupling to an external bath. *The Journal of Chemical Physics* **1984**, *81*, 3684–3690.

(S4) Swope, W. C.; Andersen, H. C.; Berens, P. H.; Wilson, K. R. A computer simulation method for the calculation of equilibrium constants for the formation of physical clusters of molecules: Application to small water clusters. *The Journal of Chemical Physics* **1982**, *76*, 637–649.
